# Compositional Shift of Bacterial, Archaeal, and Fungal Communities Is Dependent on Trophic Lifestyles in Rice Paddy Soil

**DOI:** 10.3389/fmicb.2021.719486

**Published:** 2021-09-01

**Authors:** Hyun Kim, Jongbum Jeon, Kiseok Keith Lee, Yong-Hwan Lee

**Affiliations:** ^1^Department of Agricultural Biotechnology, Seoul National University, Seoul, South Korea; ^2^Interdisciplinary Program in Agricultural Genomics, Seoul National University, Seoul, South Korea; ^3^Research Institute of Agriculture and Life Sciences, Seoul National University, Seoul, South Korea; ^4^Center for Fungal Genetic Resources, Seoul National University, Seoul, South Korea; ^5^Plant Genomics and Breeding Institute, Seoul National University, Seoul, South Korea; ^6^Plant Immunity Research Center, Seoul National University, Seoul, South Korea

**Keywords:** soil microbiota, soil nutrients, microbial trophic lifestyle, random forest model, microbial association

## Abstract

The soil environment determines plants’ health and performance during their life cycle. Therefore, ecological understanding on variations in soil environments, including physical, chemical, and biological properties, is crucial for managing agricultural fields. Here, we present a comprehensive and extensive blueprint of the bacterial, archaeal, and fungal communities in rice paddy soils with differing soil types and chemical properties. We discovered that natural variations of soil nutrients are important factors shaping microbial diversity. The responses of microbial diversity to soil nutrients were related to the distribution of microbial trophic lifestyles (oligotrophy and copiotrophy) in each community. The compositional changes of bacterial and archaeal communities in response to soil nutrients were mainly governed by oligotrophs, whereas copiotrophs were mainly involved in fungal compositional changes. Compositional shift of microbial communities by fertilization is linked to switching of microbial trophic lifestyles. Random forest models demonstrated that depletion of prokaryotic oligotrophs and enrichment of fungal copiotrophs are the dominant responses to fertilization in low-nutrient conditions, whereas enrichment of putative copiotrophs was important in high-nutrient conditions. Network inference also revealed that trophic lifestyle switching appertains to decreases in intra- and inter-kingdom microbial associations, diminished network connectivity, and switching of hub nodes from oligotrophs to copiotrophs. Our work provides ecological insight into how soil nutrient-driven variations in microbial communities affect soil health in modern agricultural systems.

## Introduction

The ongoing rapid increase in the world’s population necessitates improvements in crop productivity. Crop productivity is determined by the climate, water content, available nutrients, and biological factors. Soil fertility can be modulated anthropogenically to enhance crop productivity. The Green Revolution helped to improve crop productivity by affecting controllable factors related to crop plants. The use of chemical fertilizers and pesticides improves crop productivity by providing sufficient nutrients and protection against insect pests and microbial pathogens. However, agrochemicals promote soil degradation by increasing the salinity and acidity of soils and decrease the biodiversity of agricultural environments (Matson, 1997), hampering sustainability. To improve both crop performance and environmental quality, precision agriculture (site-specific crop management) has been proposed (McBratney et al., 2005). In precision agriculture, edaphic variables (such as topography, organic matter content, moisture levels, nitrogen levels, and other factors) are measured for intra- and inter-field comparisons. However, soil microbial communities, key component of soil properties, are infrequently considered among measurable edaphic factors.

Soil microbial communities are key to the cycling of carbon, nitrogen, and other inorganic nutrients (Jansson and Hofmockel, 2018). These roles of the soil microbial communities are crucial for maintaining soil health and ecological functions. Soil microbial communities govern the turnover time of nutrient reservoir such as organic matter (Rousk and Bengtson, 2014). Soil fungal communities regulate the balance of carbon and nutrients (Žifčáková et al., 2016), and mediate phosphorus (P) and nitrogen (N) cycles by converting organic P and N compounds to mineral forms (Treseder and Lennon, 2015). Soil bacterial communities affect ecological functions as well. Soil bacterial communities can produce greenhouse gases, such as methane (CH_4_), carbon dioxide (CO_2_), and nitrous oxide (N_2_O), while decomposing organic matter, consequently affecting land atmosphere carbon exchange (Bardgett et al., 2008; Oertel et al., 2016). Soil microbial communities also provide the initial source material for plant microbiomes. Specifically, the bulk soil microbiome is the origin of the rhizosphere microbiome contributing to the initial colonization of plants (Edwards et al., 2015). Small variations in the composition and function of the initial soil microbiome can predetermine plant survival during threat of plant disease (Wei et al., 2019).

Microbial communities are sensitive to alteration in soil physical and chemical properties (Karhu et al., 2014). To understand the prevalence, distribution, and responses of microbial communities to changes in environmental conditions, life strategy concepts, such as r- and k-strategist (Andrews and Harris, 1986) and competitive- stress tolerator-ruderals (C-S-R) framework (Grime and Pierce, 2012), have been proposed. One of the proposed ecological life strategies is microbial trophic lifestyle also known as copiotrophy–oligotrophy continuum. Microbial trophic lifestyles are categorized as oligotrophy and copiotrophy according to their nutrient adaptation mode. Oligotrophs prefer low-nutrient conditions, whereas copiotrophs thrive under high-nutrient conditions (Ho et al., 2017). Previous studies reported that changes in soil bacterial communities in response to presence of recalcitrant or labile carbon substrates (Goldfarb et al., 2011) and temperature sensitivity of CO_2_ flux (Bai et al., 2017) can be explained by the copiotrophy–oligotrophy continuum. Another study also showed that ecological attributes of specific bacterial taxa can be predicted by microbial trophic lifestyle (Fierer et al., 2007). Thus, investigation of microbial trophic lifestyles will give ecological insights on the influence of soil nutrient status on the diversity and stability of the initial soil microbiome.

Rice paddy soils provide a unique environment compared to other agricultural fields. During rice cultivation, alternating dry and submerged conditions results in alternately oxidized and reduced environments (Liesack et al., 2000). This fluctuation enables the coexistence of aerobic and anaerobic microbes that play crucial roles in nutrient cycling by mediating methane production and consumption (Liesack et al., 2000), nitrification (Jiang et al., 2015), and denitrification (Arth et al., 1998). These microbial activities impact both the agricultural environment and productivity (Gao et al., 2016). The factors that influence microbial functions and communities in flooded paddy soils are of great interest. Soil properties play a role in shaping microbial community composition in paddy soils. For example, soil pH is associated with the diversity and composition of bacterial and fungal communities (Jiang et al., 2016). In addition, soil ions, such as iron and sulfate, influence the composition of bacterial and archaeal communities (Sun et al., 2018). Changes in soil moisture and redox potential significantly affect bacterial community composition (Li et al., 2021). Management activities also affect microbial community composition, diversity, and enzymatic activities during the cropping season (Jiang et al., 2016; Liu et al., 2020; Carlos et al., 2021). Although the investigation of pre-season soil conditions is important for determining how agricultural fields are managed during the cropping season, abiotic and biotic conditions in pre-season soils are less well understood than conditions during the cropping season in rice paddy soils.

In the present study, we aimed to (1) examine effects of abiotic environments on soil bacterial and fungal composition and diversity in pre-season soils, (2) find effects of soil nutrients and fertilization regimes on the distribution of microbial taxa or operational taxonomic units (OTUs), and (3) investigate their effects on microbial networks. We examine the abiotic and biotic properties of pre-season soil samples collected from 18 geographically separated rice paddies over 2 years. We demonstrate the soil nutrient-driven distribution of microbial trophic lifestyles (oligotrophs and copiotrophs) in the bacterial, archaeal, and fungal communities. Our findings help to fill a major knowledge deficiency in the ecology of soil microbial communities before cropping season.

## Materials and Methods

### Soil Collection

To compare the effects of edaphic factors and cultural practices on soil microbial communities, fields located in 9 sites were differentially managed. In total, 9 kg nitrogen, 4.5 kg phosphate, and 5.7 kg potassium per 1,000 m^2^ were applied 3 times per cultivation period in fields managed by conventional farming (CF) and without pesticides (NP). In unfertilized (non-fertilized) fields (NF), no fertilizer was applied during rice cultivation. The size of fields varied in sites (300–900 m^2^). Sampling area was restricted to 100 m^2^ (10 m × 10 m) to reduce spatial variation among fields, and further divided into 9 square-shaped sections. Soil samples were collected from the center of each section. A total of 162 soil samples were collected from the 18 fields in April 2017. In April 2018, a total of 90 soil samples were collected from 10 fields, which were identical to those of the previous year (Supplementary Figure 1 and Supplementary Table 1). In both years, fields were not plowed before sampling. To avoid the effects of plant debris, soil below a depth of 5 cm was removed. Two kg of soil below a depth of 15 cm (unplanted bulk soil) was collected from each field. Then, 500 g of each soil was used to analyze soil texture (contents of sand, silt, and clay) and chemical properties [pH, soil organic matters (SOM), total nitrogen (TN), cation exchange capacity (CEC), exchangeable Ca^2+^, Mg^2+^, Na^+^, K^+^, and phosphate (P_2_O_5_)]. To measure soil pH, 5 g of air-dried and sieved soils were added into 25 ml of deionized water and mixed for 30 min. Soil suspension was incubated for 1 h at room temperature. pH was measured using a pH meter (HM-30R, DKK-TOA, Japan). SOM and TN contents were measured following Walkley-Black methods (Walkley and Black, 1934) and Kjeldahl method (Bremner, 1960), respectively. CEC were quantified using ammonium acetate (NH_4_OAc) method (Schollenberger and Simon, 1945). To quantify contents of exchangeable cations, 5 g of air-dried soils were added into 50 ml of 1N NH_4_OAc (pH 7.0). After incubation for 30 min, the contents of cations were measured using inductively coupled plasma (ICP) emission spectroscopy (ICP-730-ES, Varian, United States; ICP-7510, Shimadzu, Japan; ICP-7400, Thermo Fisher Scientific, United States). The contents of P_2_O_5_ were measured following Bray No.1 method (Bray and Kurtz, 1945). Edaphic factors were analyzed in the National Instrumentation Center for Environmental Management (NICEM) at Seoul National University, Korea. 500 g out of 1.5 kg of collected soil was sieved through a 2 mm mesh to remove plant debris and particles larger than sand grains.

### Sample Preparation and DNA Extraction

Sieved soils (0.5 g) were transferred to Lysing Matrix E tubes from FastDNA SPIN Kit for Soil (MP Biomedicals, United States). A total of 252 samples were prepared and pulverized with a bead beater (Biospec, United States) at 4,000 rpm for 2 min. Bead beating was repeated after samples cooled in ice for 1 min. Soil DNA was extracted following the instructions of the manufacturer. The concentration of DNA in each sample was quantified with a NanoDrop^TM^ spectrophotometer (Thermo Fisher Scientific, United States). Extracted DNA was stored at –20°C until amplicons were generated.

### PCR Amplification and Sequencing

16S ribosomal RNA (16S rRNA) and internal transcribed spacer (ITS) gene sequencing were performed in a two-step PCR amplification protocol. The V4 regions of bacterial and archaeal 16S rRNA genes and the fungal ITS2 regions of nuclear rRNA genes were amplified using universal 515F/806R primers (Caporaso et al., 2011) and ITS3/ITS4 primers (White et al., 1990), respectively. Each sample was amplified in triplicate in a 25 μl reaction tube containing 12.5 μl of 2x PCR i-StarTaq^TM^ Master mix Solution (Intron Biotechnology, Korea), 0.4 μM for each forward and reverse primers, and 0.8 μM of diluted DNA template. For the ITS libraries, the conditions were the same as for the preparation of the 16S libraries. PCR was performed in the program set at initial denaturing at 98°C for 3 min, followed by 32 cycles of denaturing at 98°C for 10 s, primer annealing at 55°C for 30 s and extension at 72°C for 60 s. For ITS PCR amplification, the program was the same. Each library was accompanied by negative PCR controls to ensure that the reagents were free of contaminant DNAs. Amplicon replicates were pooled, then purified using MEGAquick-spin^TM^ Plus DNA Purification Kit (Intron Biotechnology, Korea) with additional ethanol clean-up step to remove PCR reagents and resulting primer dimers completely. The second PCR was done with the Nextera XT Index Kit (Illumina, United States). DNA templates were diluted to equal concentrations after being measured by the Infinite 200 pro (TECAN, Switzerland). The libraries were then pooled into equal concentrations into a single library and concentrated using AMPure beads (Beckman Coulter, United States). The pooled library then went through a final gel purification stage to remove any remaining unwanted PCR products. Pooled libraries were sequenced using the Illumina MiSeq platform with 2 × 300 base pair read length. The sequencing was done in the National Instrumentation Center for Environmental Management (NICEM) at Seoul National University, Korea.

### Processing and Filtering of 16S rRNA and ITS Sequences

The sequenced reads were processed with QIIME2 (version 2018.6) (Bolyen et al., 2019). After demultiplexing, the resulting sequences were merged with PEAR (Zhang et al., 2014) and then quality filtered using the command denoise-single implemented in the DADA2 plugin (Callahan et al., 2016) in the QIIME2 (version 2018.6) pipeline. The high-quality sequences were clustered into OTUs based on 97% sequence similarity using the open reference vsearch algorithm (vsearch cluster-features-open-reference) (Rognes et al., 2016) against the Silva 99% OTU representative sequence database (v132, April 2018) (Quast et al., 2012) and then assembled into an OTU table. Bacterial OTUs were chimera filtered using the vsearch uchime-*de novo* algorithm (Edgar et al., 2011). Fungal OTUs were checked for chimeric sequences using Uchime-ref algorithm against the dedicated chimera detection ITS2 database (June 2017 version) (Nilsson et al., 2015). The taxonomy of the non-chimeric OTUs was assigned using Naïve Bayes algorithm implemented in the q2-feature-classifier prefitted to the Silva database for V4 region of 16S rRNA regions (Bokulich et al., 2018). For the ITS2 region, taxonomy assignment was done with q2-feature-classifier prefitted to UNITE database (UNITE_ver7_dynamic of Jan 2017) (Nilsson et al., 2019). Bacterial sequences ranging from 200 to 300 bp long and fungal sequences ranging from 100 to 490 bp long were used for further analyses. The OTU table was imported to R by the phyloseq package (McMurdie and Holmes, 2013) for further analysis. Sequences from host DNA and OTUs unassigned at the kingdom-level were removed (bacterial OTU: orders “*Chloroplast*” and “*Rickettsiales*”; fungal OTU: Kingdoms “*Unassigned*,” “*Chromista*,” and “*Plantae*”). OTUs detected from negative controls were removed from the samples.

### Statistical Analysis and Visualization

Unless otherwise stated, all statistical analyses were performed with R version 3.4.4 (R Core Team, 2013). Statistical significance was determined at ɑ = 0.05, and where appropriate, the statistical significance was corrected for multiple hypothesis testing using the false discovery rate (FDR) method. The OTU table was normalized by cumulative-sum scaling (CSS) and log-transformed by cumNorm() from the R package metagenomeSeq (v1.24.0) (Paulson et al., 2013). Rarefaction was done when calculating alpha diversity (McMurdie and Holmes, 2014). Shannon and Simpson indices were calculated using the function alpha() in the R package microbiome (v1.9.13) (Lahti and Shetty, 2019). Two-sided Mann-Whitney U test, Kruskal-Wallis test, and Dunn’s test were all performed in R. Taxa above relative abundance of 0.5% of each sample were visualized with the R package ggplot2 (v3.2.1) (Wickham, 2016) for taxonomic composition analysis. The Bray–Curtis dissimilarity matrix was calculated to build both unconstrained and constrained Principal Coordinate Analyses (PCoA). The constrained PCoA was constrained by soil site (location), soil texture, and cultural practice, respectively, using the function capscale() implemented in the Vegan package (v2.5-5) (Oksanen et al., 2018) with the R script made available by Zgadzaj et al. (2016). Permutational multivariate analysis of variance (PERMANOVA) was conducted using the function adonis() from the Vegan package. Putative oligotrophs and copiotrophs were identified with the correlation between relative abundances of OTUs and SOM or TN levels based on Spearman’s rank correlation since SOM and TN showed significant correlations with other soil physicochemical properties (Supplementary Figure 19). OTUs showing significant positive correlation with SOM or TN (*P* < 0.05) were classified as putative copiotrophs, whereas OTUs showing significant negative correlations with SOM or TN were classified as putative oligotrophs. Linear regression between relative abundance of putative oligotrophs or copiotrophs and microbial diversity was calculated using lm() command.

### Identification of OTUs Sensitive to Fertilization Regimes

To identify OTUs associated with fertilization regimes depending on nutrient status, differentially abundant OTUs (daOTUs) were investigated. Soil samples were divided into nutrient-poor and nutrient-rich soils based on sand, clay, and SOM contents among soils in geographically identical but differently fertilized sites. Nutrient-poor soils consisted of 54 samples in CJ (non-fertilized condition (‘17, samples collected in 2017), *n* = 9; fertilized condition (‘17), *n* = 9; non-fertilized condition (‘18, samples collected in 2018), *n* = 9, fertilized condition (‘18), *n* = 9) and YS (non-fertilized condition, *n* = 9; fertilized condition, *n* = 9) fields, whereas nutrient-rich soils consisted of 36 samples of MY (non-fertilized condition, *n* = 9; fertilized condition, *n* = 9) and NJ (non-fertilized condition, *n* = 9; fertilized condition, *n* = 9) fields. A zero-inflated Gaussian distribution mixture model was used by applying the function fitZig() from metagenomeSeq. Moderated *t*-tests using the makeContrasts and eBayes commands from the R package Limma (v3.34.9) (Ritchie et al., 2015) were used to define daOTUs. Differences in abundance were considered significant at a false discovery rate (FDR)-adjusted *P* < 0.05. Differentially abundant bacterial, archaeal, and fungal OTUs were defined as daOTUs and visualized in MA plots using R package ggplot2.

### Generation of a Random Forest Classification Model

To find the most important community responses to fertilization in different soil conditions (nutrient-poor and -rich environments), random forest models were constructed. The classification model was built by setting non-fertilized and fertilized field (0 and 1, respectively) as a function of CSS-normalized abundances of OTUs using Random Forest (RF) algorithm (randomForest package, v4.6-14) (Liaw and Wiener, 2002) in the nutrient-poor (*n* = 54) and –rich environments (*n* = 36). Two-thirds of the total samples were randomly sampled as the training set. Ten-fold cross validation (caret package, v6.0-81) (Kuhn et al., 2018) were analyzed with the remaining test set in order to check the accuracy of the RF classifiers of each kingdom. The RF classifiers of the nutrient-poor environment gave cross-validation accuracy of 0.883 (bacteria), 0.95 (archaea), and 0.944 (fungi). The RF classifiers of the nutrient-rich environment gave cross-validation accuracy of 0.85 (bacteria), 0.908 (archaea), and 0.866 (fungi). OTUs were ranked by their importance in contributing to the accuracy of non-fertilized/fertilized field prediction in the RF model by calculating the mean decrease in Gini impurity. This step was done using the function importance() command in the randomForest R package. Ten-fold cross validations were performed while excluding less important OTUs to evaluate model performance as a function of inclusion of the top fertilization regime-discriminating OTUs using the rfcv() in the randomForest R package (Edwards et al., 2018). The minimum number of OTUs with the prediction error rate which is as low as the error rate of the full RF model 8,045 (bacteria), 342 (archaea), and 2,282 (fungi) in the nutrient-poor environment and 7,969 (bacteria), 259 (archaea), and 2,636 (fungi) in the nutrient-rich environment was determined. In the samples of nutrient-poor environments, there was a rapid increase in the prediction error rate when the model included approximately less than 35 (bacteria), 25 (archaea), and 35 (fungi) of the most important OTUs, which prompted the setting of the threshold to 35, 25, and 35, respectively. On the other hand, in the samples of nutrient-rich environments, there was a rapid increase in the prediction error rate when the model included approximately less than 30 of the most important OTUs, which prompted the setting of the threshold to 30. The top OTUs from the RF models of each kingdom were further categorized as non-fertilized-enriched, fertilized-enriched, or non-differential OTUs depending on the result of differential abundance test above.

### Construction and Analyses of Microbial Co-occurrence Networks

Multi-kingdom co-occurrence networks were constructed to infer variation in microbial associations according to fields and cultural practices using the method described in the previous work (Kim et al., 2020). To construct the co-occurrence networks of each field (*n* = 9), an average of 3,808 OTUs (all OTUs consisting of microbial communities in each field) were used (Supplementary Table 11). Bacterial, archaeal, and fungal OTU counts were CSS-normalized, respectively, and then merged into a single table to estimate the correlations among OTUs. The CSS-normalized multi-kingdom OTU tables were used as inputs for network construction. The SparCC algorithm was used to infer co-occurrence patterns (Watts et al., 2019). Significant correlations were defined at correlation coefficient (*r*) > 0.6 or < –0.6, and FDR-adjusted *P* < 0.05. Visualization was performed with Gephi (v0.9.2) (Bastian et al., 2009) using the Force Atlas 2 layout. The node, edge, and network properties (degree, betweenness centrality, closeness centrality, and clustering coefficients) were investigated with igraph. Hub OTUs were defined based on within-module connectivity (*Zi*) and among-module connectivity (*Pi*) relationship (Guimerà and Nunes Amaral, 2005). Based on *Zi* and *Pi*, network hubs (*Zi* > 2.5 and *Pi* > 0.62), module hubs (*Zi* > 2.5 and *Pi* < 0.62), connectors (*Zi* < 2.5 and *Pi* > 0.62), and peripherals (*Zi* < 2.5 and *Pi* < 0.62) were defined. To find microbial associations in which functional groups are involved, the distribution of taxa involved in degradation of organic compounds (*Anaerolinea*, *Phenylobacterium*, *Dechloromonas*, and *Cellulomonas*) (Lynd et al., 2002; Salinero et al., 2009; Eberspächer, 2015; Liechty et al., 2020), iron reduction (*Geobacter* and *Anaeromxyobacter*) (Wilmoth et al., 2018), syntroph (*Syntrophus* and *Syntrophobacter*) (McInerney et al., 2009), methanotroph (*Methylosarcina*, *Methylomonas*, and *Methylobacter*) (DeJournett et al., 2007), fungal saprotroph (*Guehomyces* and *Papiliotrema*) (Duarte et al., 2018), and archaeal methanogen (*Methanosarcina*, *Methanosaeta*, *Methanocella*, *Methanobacterium*) (Wen et al., 2017) were investigated.

## Results

### Soil Properties Reveal Environmental Variations Among Rice Paddy Fields

**FIGURE 1:**
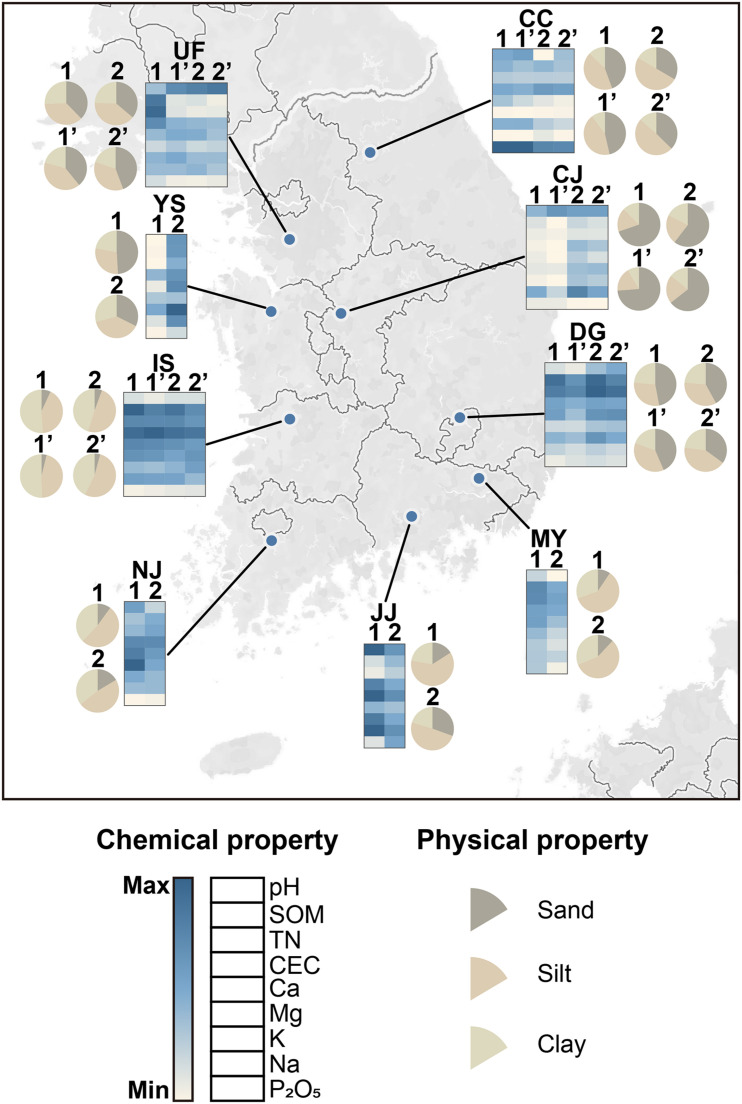
Geographic distribution of soil sampling sites and physicochemical properties of the fields. The latitudes and longitudes of the sites were visualized with Tableau Desktop (2019.4). Heat maps were constructed with Morpheus software (https://software.broadinstitute.org/morpheus/). Each square in the heat map indicates chemical properties quantified in this study. Colors indicate the relative values of the chemical properties across all fields. Pie charts show the proportions of sand, silt, and clay particles. Numbers indicate the paddy fields examined at each site. The fields investigated in 2018 are indicated by apostrophes next to the field numbers. Values of the soil physical and chemical properties are listed in Supplementary Table 1. CC, Chuncheon; CJ, Cheongju; DG, Daegu; IS, Iksan; JJ, Jinju; MY, Miryang; NJ, Naju; UF, University Farm (Suwon); YS, Yesan; SOM, soil organic matter; TN, total nitrogen; CEC, cation-exchange capacity (number of exchangeable cations per unit dry weight); P_2_O_5_, phosphate; K, potassium; Mg, magnesium; Na, sodium; Ca, calcium.

The physicochemical properties were heterogeneous among fields in both years (Figure 1 and Supplementary Table 1). Soil pH ranged from 5.1 to 7.1, which is weakly acidic to neutral. Indicators of soil fertility [ion contents, total nitrogen (TN), soil organic matter (SOM), and cation exchange capacity (CEC)] also varied among sites. Iksan (IS) soil had the highest overall fertility. Phosphate (P_2_O_5_) levels were high in Chuncheon (CC) soil in both years (201.4–271.08 mg kg^–1^). The distribution of soil particles also differed among sites (Figure 1 and Supplementary Table 1). Cheongju (CJ) had the highest sand content, whereas IS and NJ had the highest clay contents. These findings indicate that the soil environments were highly heterogeneous despite the similar ecological characteristics of the sampled rice paddies.

### Taxonomic Composition of Microbial Communities Varied Among Paddy Fields

To investigate the relationships between edaphic factors and microbial community composition, the bacterial, archaeal, and fungal communities were analyzed. In total, 13,373,658 bacterial reads, 1,124,321 archaeal reads, and 12,191,641 fungal reads were obtained. After the removal of unwanted reads (chimeras, undesirable taxa, and singletons), 33,199 operational taxonomic units (OTUs) [22,623 bacterial OTUs (bOTUs), 1,139 archaeal OTUs (aOTUs), and 9,437 fungal OTUs (fOTUs)] were identified at 97% sequence similarity. Bacterial communities were generally dominated by *Proteobacteria*, *Chloroflexi*, *Acidobacteria*, *Actinobacteria, and Bacteroidetes* (Supplementary Figure 2A). The bacterial community composition differed among locations. In the archaeal community, the phyla *Euryarchaeota* and *Crenarchaeota* dominated all samples, although archaeal diversity may be underestimated due to the use of the 515f/806r primer set (Supplementary Figure 2B). Fungal communities were dominated by *Ascomycota* and *Basidiomycota* but exhibited high variability among fields at the class level (Supplementary Figure 2C).

### Soil Physicochemical Properties Contribute to Variations in Community Composition

**FIGURE 2:**
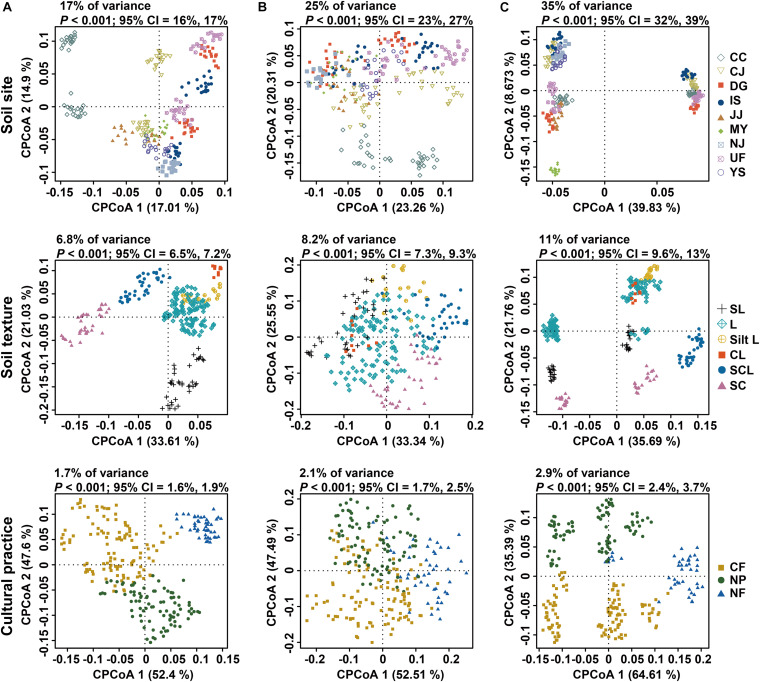
Constrained principal coordinate analysis of bacterial, archaeal, and fungal communities in rice paddy fields. Variations in **(A)** bacterial, **(B)** archaeal, and **(C)** fungal communities constrained by sampling site (upper panel), soil texture (middle panel), and cultural practice (bottom panel). Cumulative sum scaling (CSS)/log-transformed reads were used to calculate Bray–Curtis distances. Colors and shapes indicate sampling sites, soil textures, and cultural practices, respectively. CC, Chuncheon; CJ, Cheongju; DG, Daegu; IS, Iksan; JJ, Jinju; MY, Miryang; NJ, Naju; UF, University Farm (Suwon); YS, Yesan; SL, sandy loam; L, loam; Silt L, silt loam; CL, clay loam; SCL, silt clay loam; SC, silty clay; CF, conventional farming (managed with both pesticides and fertilizers); NF, no fertilizers (managed with only pesticides); NP, no pesticides (managed with only fertilizers).

A constrained principal coordinate analysis (PCoA) was conducted to investigate the effects of sampling site and soil texture on microbial community composition, because soil chemical properties varied with geographic location and were significantly correlated with soil particle type (Supplementary Tables 1, 2). When constrained by site and soil texture, all factors contributed to the clustering of all samples (Figure 2). Site and soil texture impacted the fungal community more strongly than they did the bacterial and archaeal communities. This result is consistent with variations in fungal composition (Supplementary Figure 2). Soil texture could significantly explain 6.8, 8.2, and 11% of the compositional variance in the bacterial, archaeal, and fungal communities, respectively, under constrained conditions. Unconstrained PCoA also corroborated this finding (Supplementary Figure 3). In unconstrained PCoA, the contribution of soil texture to compositional variance accounted for 9% (bacteria), 12.6% (archaea), and 9.7% (fungi) of the total variance. These results indicate that the bacterial community is less strongly impacted by soil texture than the archaeal and fungal communities. Permutational multivariate analysis of variance (PERMANOVA) was conducted to quantify the contributions of edaphic factors to community variation. The result of PERMANOVA showed significant contributions of each factor to variations in microbial community composition (all, *P* = 0.0001) (Supplementary Table 3). This conclusion was also supported by the relationships between principal coordinates (PCos) and soil properties (Supplementary Table 4). Canonical correspondence analysis (CCA) was conducted to identify the links between soil properties and microbial community composition. All factors contributed to the clustering of each community (Supplementary Figure 4). In the CCA plot, arrow lengths indicate the contributions of variables to community variation. The pH and cations (Mg^2+^, Ca^2+^, Na^+^, and K^+^) were notably linked to compositional variation in bacteria. However, pH contributed little to archaeal composition. Sand content had a stronger impact on the fungal community than bacterial or archaeal composition (Supplementary Figure 4A). This result was corroborated by PERMANOVA (Supplementary Table 3; bacteria, *R*^2^ = 0.013; archaea, *R*^2^ = 0.01; fungi, *R*^2^ = 0.022; all, *P* = 0.0001). These results suggest that even the same physical and chemical properties may have differing influences depending on the microbial community.

### Cultural Practices Shape Pre-season Soil Microbial Community Composition and Diversity

Cultural practices related to fertilizers and pesticides have varying effects on different microbial communities. Sampling sites were classified into 3 groups based on such practices: conventional farming (CF; managed with both pesticides and fertilizers), no fertilizers (NF; only pesticides), and no pesticides (NP; only fertilizers). PERMANOVA showed that cultural practices could explain 1.6, 1.4, and 2.1% of the variance in the bacterial, archaeal, and fungal communities, respectively (all, *P* = 0.0001) (Supplementary Table 3). A permutational test for homogeneity of multivariate dispersions was used to assess whether the effects of cultural practices were driven by biological differences or were artifacts of heterogeneous dispersion (Anderson, 2001). Most sites yielded non-significant *P*-values (Supplementary Table 3), suggesting that the variance by cultural practices was primarily derived from biological differences. The results of constrained PCoA also showed that fields managed with the same practices clustered together, irrespective of sites (Figure 2 and Supplementary Figure 5), suggesting that the effects of cultural practices may be consistent across sites. Richness and Shannon index values of the bacterial communities were significantly different among the three cultural practices assessed. On the other hand, the alpha diversity indices of the archaeal and fungal communities did not differ significantly (Supplementary Figure 6). Meanwhile, the distributions of bacterial diversity and soil nutrients exhibited the opposite tendency (Supplementary Figure 7). Among the 28 fields examined, 5 pairs of subsamples from non-fertilized and fertilized conditions were selected. Pairwise comparison of alpha diversity indices between non-fertilized and fertilized fields in this group showed that bacterial and fungal Shannon indices and archaeal richness scores were significantly higher under non-fertilized conditions than under fertilized conditions (Supplementary Figure 8). The PERMANOVA results showed that fertilization affects soil microbial community composition and diversity more strongly than did pesticides (Supplementary Table 3).

### Soil Physicochemical Properties Influence Microbial Abundance

As microbial community composition was affected by variations in edaphic factors, we next investigated which taxa are significantly correlated with each variable. Certain phyla were significantly correlated with each physicochemical edaphic factor (Supplementary Table 5). For example, the relative abundance of *Acidobacteria*, an oligotrophic phylum, was significantly negatively correlated with SOM (*r* = –0.2647, *P* < 0.001) and TN (*r* = –0.2066, *P* < 0.001). The relative abundances of *Cyanobacteria* (autotroph) (SOM, *r* = –0.3754; TN, *r* = –0.444; both, *P* < 0.001) and *Glomeromycota* (SOM, *r* = –0.3377; TN, *r* = –0.204; both, *P* < 0.001), which includes mycorrhizae, were also significantly negatively correlated with SOM and TN levels. On the other hand, the archaeal phylum *Crenarchaeota* was positively correlated with the same variables (SOM, *r* = 0.5468; TN, *r* = 0.5469; both, *P* < 0.001). The abundant fungal phyla, *Ascomycota* (*r* = 0.2201, *P* < 0.001) and *Basidiomycota* (*r* = 0.4416, *P* < 0.001) were both significantly positively correlated with sand content. This result may support the stronger impact of sand content on fungal composition than on bacterial and archaeal communities, as shown in the CCA results (Supplementary Figure 4A). Significant correlations between microbial abundance and edaphic factors were also obtained at the genus level (Supplementary Table 5). Among 2,669 bacterial genera detected, 1,574 genera exhibited significant correlations with at least one soil physicochemical factor. Similar to bacteria, the relative abundances of 288 of 580 fungal genera and 73 of 116 archaeal genera were significantly correlated with edaphic factors. For example, *Anaerolinea*, a bacterial genus, was significantly positively correlated with SOM (*r* = 0.285, *P* < 0.001), TN (*r* = 0.2798, *P* < 0.001), clay (*r* = 0.4269, *P* < 0.001), CEC (*r* = 0.3424, *P* < 0.001), Na^+^ (*r* = 0.2667, *P* < 0.001), K^+^ (*r* = 0.1692, *P* < 0.01), Ca^2+^ (*r* = 0.2222, *P* < 0.001), and Mg^2+^ (*r* = 0.3709, *P* < 0.001). Conversely, *Mesorhizobium*, a nitrogen-fixing bacterial genus, was negatively correlated with soil nutrients (SOM: *r* = –0.3156; TN: *r* = –0.2967; Mg^2+^: *r* = –0.2643; all, *P* < 0.001), silt (*r* = –0.1876, *P* < 0.01), and clay (*r* = –0.2901, *P* < 0.001). Among fungal saprotrophs, the abundance of Trichoderma was positively correlated with SOM (*r* = 0.3146, *P* < 0.001) and TN (*r* = 0.4136, *P* < 0.001) and negatively correlated with levels of Mg^2+^ (*r* = –0.3779, *P* < 0.001) and Na^+^ (*r* = –0.2846, *P* < 0.001), whereas that of *Guehomyces*, a basidiomycotal saprotroph, was significantly positively correlated with pH (*r* = 0.3429, *P* < 0.001) and sand (*r* = 4447, *P* < 0.001) levels. Of the genera that exhibited significant correlations with edaphic factors, the relative abundances of 592 bacterial, 116 fungal, and 36 archaeal genera were significantly affected by soil nutrients, as represented by SOM and TN. The alpha diversities of the bacterial, archaeal, and fungal communities were also significantly correlated with soil properties. The Shannon and Simpson indices of the bacterial community were significantly negatively correlated with soil nutrients (SOM: Shannon, *r* = –0.332, *P* = 6.682E-08; Simpson, *r* = –0.25, *P* = 5.883E-05; TN: Shannon, *r* = –0.305, *P* = 7.332E-07) and clay (Shannon, *r* = –0.165, *P* = 0.008; Simpson, *r* = –0.243, *P* = 9.508E-05). Archaeal diversity was also significantly negatively correlated with soil nutrients (SOM: Shannon, *r* = –0.165, *P* = 0.008; Simpson, *r* = –0.364, *P* = 2.354E-09; TN: Simpson, *r* = –0.2803, *P* = 6.206E-06) and clay (Shannon, *r* = –0.365, *P* = 2.115E-09; Simpson, *r* = –0.506, *P* = 0) similar to bacterial diversity. However, the fungal Shannon index was positively correlated with soil nutrients (SOM: *r* = 0.199, *P* = 0.0014; TN: *r* = 0.1908, *P* = 0.002) and clay (*r* = 0.267, *P* = 1.73E-05) (Supplementary Figure 9 and Supplementary Table 6). Meanwhile, pH was not significantly correlated with bacterial, archaeal, or fungal diversity indices. These results suggest that the levels of carbon and nitrogen sources may be crucial factors for the abundances and diversity of soil microbial communities.

### Trophic Lifestyles Show Differing Responses of Microbial Communities to Soil Nutrients

**FIGURE 3:**
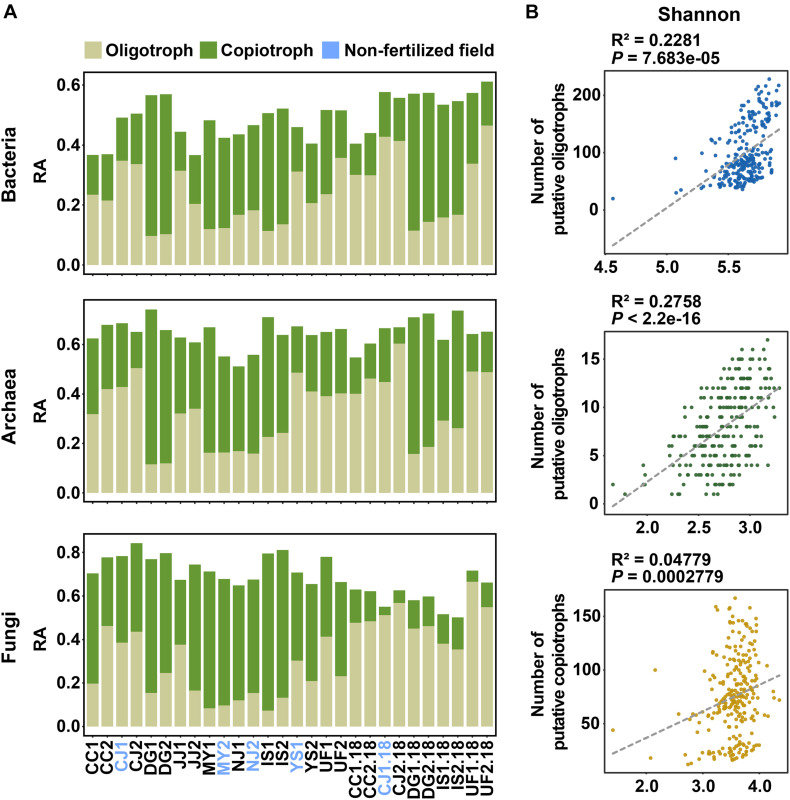
Distributions of putative oligotrophs and copiotrophs in bacterial, archaeal, and fungal communities and their relationships with microbial diversity. **(A)** Cumulative relative abundances of putative oligotrophs and copiotrophs in rice paddy fields. Putative oligotrophs and copiotrophs were classified based on the correlations between OTU abundances and SOM or TN content. Putative oligotrophic OTUs were defined as OTUs that had a significant negative correlation with SOM or TN. By contrast, putative copiotrophs were identified as OTUs with a significant positive correlation to SOM or TN. Colors indicate trophic lifestyles (light green, oligotrophy; dark green, copiotrophy). **(B)** Relationship between the Shannon index and the number of putative oligotrophs or copiotrophs in bacterial (upper panel), archaeal (middle panel), and fungal (bottom panel) communities. The relationships were estimated using linear regression. Dashed lines indicate trend lines for each plot. RA, relative abundance.

Since we found that soil nutrients (SOM and TN) and fertilization significantly influence the diversity of microbial communities, we hypothesized that microbial trophic lifestyles (oligotrophy and copiotrophy) could be related to variations in communities. Oligotrophs can grow in low-nutrient conditions, whereas copiotrophs require high levels of nutrients. Based on this definition, we classified oligotrophs and copiotrophs based on the correlations between the relative abundance of taxa or OTUs and SOM or TN content as carbon or nitrogen resource (Ho et al., 2017) (Supplementary Table 5). Putative oligotrophs were defined as OTUs that had significant negative correlations with SOM or TN, whereas putative copiotrophs were defined as OTUs that had significant positive correlations with soil nutrients. We found 1,830 putative oligotrophs (1,120 bOTUs, 53 aOTUs, and 657 fOTUs) and 1,733 putative copiotrophs (954 bOTUs, 81 aOTUs, and 698 fOTUs) among a total of 33,199 OTUs (Supplementary Table 7). The relative abundances of OTUs with the two trophic lifestyles varied among sampling sites (Figure 3A and Supplementary Table 7). In bacterial and archaeal communities, the proportion of oligotrophs overwhelmed copiotrophs in low-nutrient soil conditions. Linear regression showed that the numbers of putative oligotrophs in bacterial and archaeal communities contributed substantially to the Shannon and Simpson index values, respectively (Figure 3B). On the other hand, fungal diversity was governed by the numbers of putative copiotrophs (Figure 3B). These results may partially explain the significant negative correlations of prokaryotic diversity with soil nutrients and significant positive correlation of fungal diversities with soil nutrients (Supplementary Figure 9 and Supplementary Table 6). When considering all samples, the numbers and relative abundances were significantly higher for oligotrophs under non-fertilized conditions than under fertilized conditions in bacterial communities (Supplementary Figure 10). However, for the archaeal community, there were no significant differences in the number and relative abundances of oligotrophs and copiotrophs among different cultural practices (Supplementary Figure 10). For the fungal community, the only significant difference among the three cultural practices was in the number of oligotrophs (Supplementary Figure 10). Significant differences in the number of oligotrophs and copiotrophs were also found between fertilized and non-fertilized fields (Supplementary Figures 11, 12). The numbers of bacterial and fungal oligotrophs were significantly higher in non-fertilized fields showing low soil nutrient levels (CJ and YS). Meanwhile, significant differences were found in the number of bacterial copiotrophs between fertilized and non-fertilized conditions in fields with relatively high soil nutrient levels (MY and NJ). These results suggest that the same fertilization regime may have differing ecological effects on the distributions of oligotrophs and copiotrophs according to endemic soil conditions.

### Random Forest Models Reveal the Effects of Fertilization on OTU Abundance Patterns

To identify OTUs related to the soil condition-dependent effects of fertilization on microbial communities, differentially abundant OTUs (hereafter, daOTUs) associated with fertilization were investigated from nutrient-poor (CJ and YS) and nutrient-rich environments (MY and NJ). In total, 3,474 and 3,656 daOTUs were identified in nutrient-poor and nutrient-rich soils, respectively (Supplementary Figure 13 and Supplementary Table 8). Putative oligotrophs and copiotrophs sensitive to fertilization accounted for 2.5–5.3% of the total daOTUs. Putative oligotrophs in prokaryotic communities were significantly affected by fertilization under nutrient-poor conditions, whereas putative copiotrophs in all communities were sensitive under nutrient-rich conditions (Supplementary Table 8). Beneficial microbes and pathogens were found in varying frequencies among phylogenetic groups. Among putative beneficial microbes, *Glomeromycota* (5 OTUs in nutrient-rich environments, 3 OTUs in nutrient-poor environments) was enriched under non-fertilized conditions. OTUs of *Rhizobium*, *Mesorhizobium*, and *Bradyrhizobium* were enriched under both non-fertilized and fertilized conditions of nutrient-poor environments. However, OTUs of those three genera were also enriched under non-fertilized conditions in nutrient-rich environments, but not under fertilized conditions in nutrient-rich environments. For putative pathogens, OTUs belonging to *Fusarium* were significantly abundant under both non-fertilized and fertilized conditions in nutrient-poor and nutrient-rich environments. On the other hand, OTUs of *Nigrospora* and *Cladosporium* were significantly abundant in non-fertilized nutrient-poor environments and fertilized nutrient-rich environments.

**FIGURE 4:**
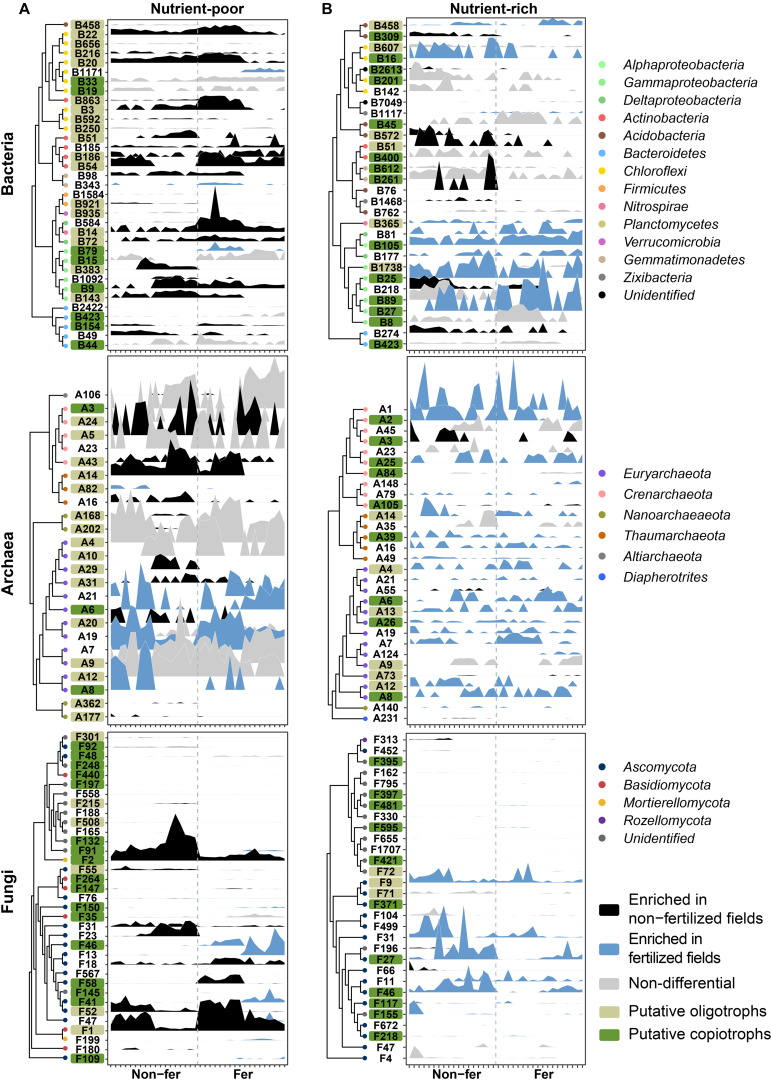
Relative abundance profiles of fertilization-responsive OTUs in nutrient-poor and nutrient-rich environments from random forest classification. OTUs are colored based on their categorization as “non-fertilized field-enriched,” “fertilized field-enriched,” and “non-differential” according to the results of the differential abundance test presented in Supplementary Figure 13. The RF models for each community in **(A)** nutrient-poor and **(B)** nutrient-rich environments were constructed using a 10-fold cross validation method. OTUs were ranked based on their contribution to the accuracy of prediction in the RF model, which was determined by calculating the mean decrease in Gini impurity. OTUs are ordered along the *y*-axis based on phylogenetic relationships among OTUs. Each tick on the *x*-axis indicates an individual sample of non-fertilized and fertilized fields. Non-fer, non-fertilized fields; Fer, fertilized fields.

To find the most important OTUs associated with fertilization under different soil conditions, we constructed random forest (RF) models in nutrient-poor and nutrient-rich environments. Using RF models, the top 25–35 OTUs were selected as they reflected the same cross-validation error rate for RF models of bOTUs, aOTUs, and fOTUs (Figure 4 and Supplementary Tables 9, 10). Important OTUs were indexed based on their enrichment-depletion patterns between non-fertilized and fertilized fields (Supplementary Figure 13 and Supplementary Table 8). Most of the important OTUs belonged to the dominant phyla in their microbial communities. In the bacterial community, *Alphaproteobacteria* OTUs were solely found in nutrient-rich environments. In the fungal community, OTUs of Basidiomycota were exclusively detected in nutrient-poor environments (Figure 4). The enrichment patterns revealed different community responses to the same fertilization regime in different environments. For example, in nutrient-poor environments, the depletion of prokaryotic oligotrophs and enrichment of fungal copiotrophs were the dominant responses (Figure 4A). In nutrient-rich environments, both putative oligotrophs and copiotrophs in the archaeal community were enriched under the fertilized conditions (Figure 4B). In the bacterial and fungal communities, enrichment of putative copiotrophs was revealed as important responses to fertilization. These results suggest that microbial trophic lifestyles tend to shift from oligotrophy to copiotrophy in nutrient-poor environments, whereas copiotrophy increases in nutrient-rich environments.

### Fertilization Influences Microbial Associations

**FIGURE 5:**
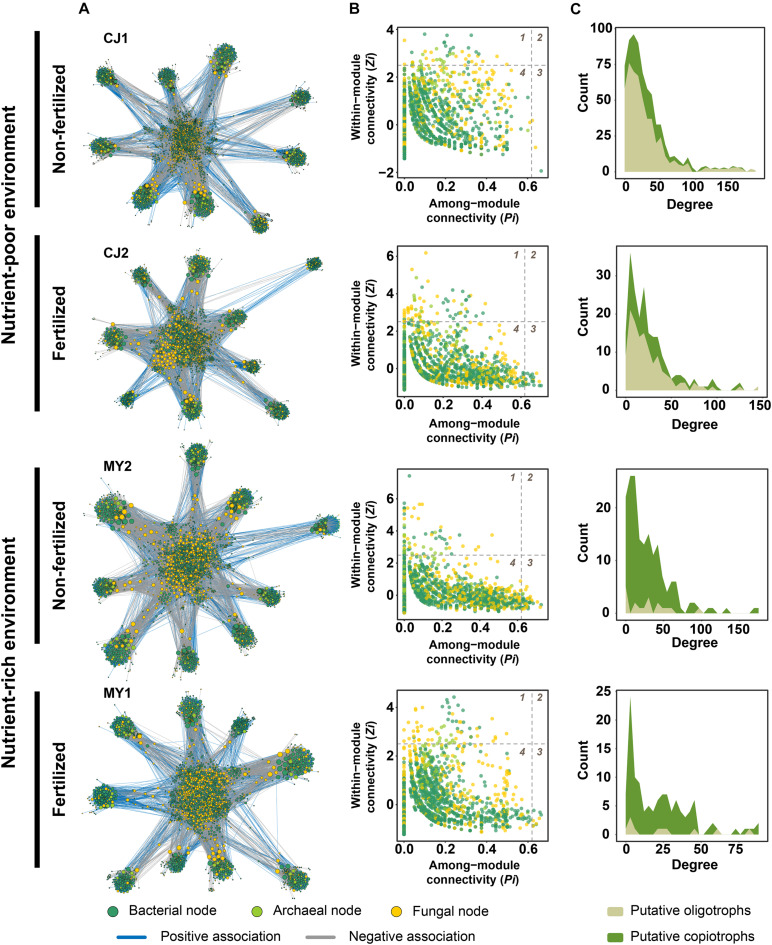
Microbial networks and hub nodes of soil microbial communities under non-fertilized and fertilized conditions in nutrient-poor and nutrient-rich environments. **(A)** Co-occurrence network of microbial OTUs detected in fields managed with different fertilization regimes in nutrient-poor and nutrient-rich environments. Each node corresponds to an OTU, and edges between nodes correspond to either positive (blue) or negative (gray) correlations inferred from OTU abundance profiles using the SparCC method (*P* < 0.05, correlation values < –0.6 or > 0.6). OTUs belonging to different microbial kingdoms are indicated with colors (Bacteria, green; Archaea, light green; Fungi, yellow), and the node size reflects their degree centrality. **(B)** Hub OTUs of microbial networks under each condition. Dashed lines indicate thresholds estimated using within-module connectivity (*Zi*) and among-module connectivity (*Pi*) values in which the roles of nodes are discriminated. The numbers of the quadrants indicate the roles of the nodes in the networks. 1, module hub (*Zi* > 2.5 and *Pi* < 0.62); 2, network hub (*Zi* > 2.5 and *Pi* > 0.62); 3, connector (*Zi* < 2.5 and *Pi* > 0.62); 4, peripheral (*Zi* < 2.5 and *Pi* < 0.62). **(C)** Distribution of degrees of putative oligotrophs and copiotrophs in microbial networks. The degree indicates the number of associations (edges) shared by each node in a network.

Based on the results of RF modeling, we hypothesized that microbial associations in each environment are also differently affected by fertilization. To test this hypothesis, we constructed microbial networks for the non-fertilized and fertilized fields (Figure 5A and Supplementary Figure 14). The average microbial network for non-fertilized fields consisted of 2,296 nodes and 31,162 edges, whereas that for fertilized fields had 2,071 nodes and 26,964 edges (Supplementary Table 11). Greater bacterial node numbers were observed in co-occurrence networks for non-fertilized fields. The proportion of negative associations increased for fertilized fields (Supplementary Figure 15). However, no significant difference in the degree, which is the number of edges connected with each node, was observed (Supplementary Figure 16). Despite the lack of significant differences in degree between non-fertilized and fertilized fields, non-fertilized fields were associated with more complex networks than were fertilized fields based on the numbers of network edges and nodes. Network complexity was compared quantitatively using complexity index B (*B*), Bertz complexity index, and distance degree/code centric indices. The complexity of networks for non-fertilized fields (CJ1, *B* = 8.102197; CJ1 (′18), *B* = 5.832945; YS1, *B* = 8.935147; MY2, *B* = 8.869343; NJ2, *B* = 9.174037) was slightly higher than that for fertilized fields (CJ2, *B* = 8.061109; CJ2 (′18), *B* = 0; YS2, *B* = 8.520694; MY1, *B* = 7.648643; NJ1, *B* = 9.145589). The Bertz complexity and distance degree/code centric indices consistently indicated higher complexity in non-fertilized fields (Supplementary Table 11).

Hub nodes also indicated that fertilization affects microbial communities. Hub nodes were based on within-module connectivity (*Zi*) and among-module connectivity (*Pi*) (Figure 5B). No network hubs were found, but module hubs and connectors were identified (Supplementary Table 12). Most of the bacterial module hub nodes were *Proteobacteria*, *Acidobacteria*, *Actinobacteria*, and *Chloroflexi*. Fungal module hubs were *Ascomycota* and *Basidiomycota*. Archaeal module hubs consisted of *Euryachaeota* and *Crenarchaeota*. Although the hub node composition differed for each field, the trophic lifestyle distribution of hub nodes showed that fertilization affects microbial associations. In both nutrient-poor and nutrient-rich environments, the number of oligotrophic module hubs (Student’s *t*-test, *P* = 0.0481) and their associated edges (Student’s *t*-test, *P* = 0.0303) decreased significantly under fertilized conditions. The numbers of copiotrophic hubs and their associated edges increased in fertilized fields, but this change was not significant (Student’s *t*-test, *P* = 0.3851 for number of hubs; *P* = 0.4283 for number of edges). These results suggest that hub nodes tend to consist of oligotrophs for non-fertilized fields, whereas copiotrophs are more central in networks for fertilized fields due to the reduction in oligotrophic hub nodes (Supplementary Table 12). Trophic lifestyles contributing to network structures also changed from oligotrophs to copiotrophs with the shift from non-fertilized to fertilized fields (Figure 5C and Supplementary Figure 17). These changes were more obvious in nutrient-poor environments. This finding suggests that changes in trophic lifestyles might be involved in the decreased degree centrality of fertilized fields, despite non-significant differences relative to non-fertilized fields. Interestingly, archaeal nodes exhibited significantly higher mean degree and betweenness centrality values than did bacterial and fungal nodes in spite of low numbers in all microbial networks (Supplementary Figure 18). Methanogenic archaea were significantly associated with bacteria involved in bacterial decomposers (*Anaerolinea*, *Phenylobacterium*, *Cellulomonas*, and *Dechloromonas*), iron reduction (*Geobacter* and *Anaeromyxobacter*), syntrophy (*Syntrophus* and *Syntrophobacter*), and methanotrophy (*Methylosarcina*, *Methylomonas*, and *Methylobacter*) (Supplementary Table 13). We also found that methanogenic archaea were associated with fungal saprotrophs belonging to *Guehomyces* and *Papiliotrema* (Supplementary Table 13). These results suggest that archaeal nodes might contribute to rice paddy soil networks by mediating diverse associations with moderate connectivity.

## Discussion

Soil is a fundamental environment where organic matter, such as manure and plant debris, is recycled. Variations in soil type, landscape characteristics, and cultural practices affect biotic and abiotic factors involved in ecological recycling, resulting in differing nutrient distributions. We analyzed the bacterial, archaeal, and fungal communities in 252 soil samples from 18 paddy fields managed under a consistent cultural practice for at least 5 consecutive years. Our assessment of various geographic locations and years revealed extensive blueprints of paddy soil microbial communities. Our approach also allowed us to explore the ecological effects of natural soil conditions and fertilizers on community diversity and multi-kingdom associations.

We revealed the various compositions of bacterial, archaeal, and fungal communities in pre-season soils. *Proteobacteria*, *Chloroflexi*, *Acidobacteria*, and *Actinobacteria* were the dominant bacterial phyla in pre-season soils (Supplementary Figure 2A). The presence of several aerobic taxa suggests oxic conditions in pre-season rice paddy soils. For instance, *Sphingomonas*, a strictly aerobic bacterial genus, was dominant. In the fungal community, *Guehomyces* (a pectinolytic yeast under oxic conditions) (Cavello et al., 2017) and *Mortierella* dominated the examined soils (Supplementary Figure 2C). *Hapholoma*, which can produce methane under oxic conditions (Lenhart et al., 2012), further confirms the aerobic status of the soils. The soil microbial communities also harbored putative plant-associated microbes. For example, fungal communities included putative plant pathogens of rice (*Curvularia*, *Magnaporthe*, and *Sarocladium*), as well as growth-promoting fungi such as arbuscular mycorrhizae (*Glomeromycota*) and dark septate endophytic fungi (*Periconia*) (Li et al., 2018). Methanogenic archaea that can colonize the rice root endosphere (*Methanobacterium*) and rhizosphere (*Rice Cluster I* and *Methanosarcina*) (Edwards et al., 2015) were also detected (Supplementary Figure 2B). These results suggest that pre-season soils harbor soil microbial communities that can be detected during cropping season, although it remains unclear whether these communities are active or dormant.

The distribution of archaeal communities showed the specificity of the paddy soil environment compared to other agricultural soils. The archaeal communities of rice paddy fields were reportedly dominated by *Euryarchaeota*, whereas *Thaumarchaeota* was enriched in dryland soils with history of maize cultivation (Jiao et al., 2019). This finding suggests that the archaeal composition of pre-season soils might be more similar to that of flooded bulk soil than to aerobic dryland soil (Supplementary Figure 2B). Given that anaerobic methanogenic archaea and *Verrucomicrobia*-like strains are tolerant of oxygen and desiccation (Liesack et al., 2000), this characteristic enables anaerobic microbes to survive during dry and aerobic periods. These results suggest that soil domestication (Edwards et al., 2019) and repeated environmental filtering during the growing season (Jiao et al., 2019) could shape paddy soil-specific microbial communities.

Another finding presents that soil nutrients influence the diversity of microbial communities by affecting the distribution of oligotrophs and copiotrophs. Bacterial and archaeal diversities were negatively correlated with soil nutrients, whereas fungal diversity was positively correlated with soil nutrients (Supplementary Figure 9). Putative oligotrophs and copiotrophs are involved in these contrasting responses of the prokaryotic and fungal communities to soil nutrients (Figure 3), which may be affected by differences in nutrient accessibility among soil microbial communities. Fungi can better access labile compounds than bacteria and archaea due to their mycelial networks (Boddy, 1999). Plant residues support fungal saprotrophs that efficiently degrade plant-derived recalcitrant organic matter into labile substrates (Van Der Wal et al., 2013). The ubiquitous distribution of pectinolytic and cellulolytic fungi, such as *Guehomyces* (Cavello et al., 2017), *Solicoccozyma* (Mašínová et al., 2017), *Schizothecium* (Demoor et al., 2019), and *Papiliotrema* (Kim et al., 2018), corroborates the predominance of putative fungal saprotrophs in the examined soils. This might be related to the presence of rice straw (consisting of cell wall-derived polysaccharides, including cellulose, hemicellulose, lignin, and pectin (Ni’matuzahroh et al., 2019)) as a major carbon source.

Our study revealed that fertilization exerted a stronger effect on microbial communities than pesticides. Bacterial and fungal community diversities were higher in non-fertilized fields (treated with only pesticides) than in fertilized fields (treated with only fertilizers) (Supplementary Figure 6). Given that pesticides are generally applied to the plant canopy rather than to the soil, a dilution effect might occur. Microbial diversity could be restored after pesticide application to soil (Rajbongshi et al., 2014), suggesting that microbial communities might recover over time after the application of pesticides during the non-cropping period. We also found that the switching of trophic lifestyles by fertilization differed depending on endemic soil conditions. RF modeling revealed that the depletion of putative oligotrophs and the enrichment of putative copiotrophs are important for prokaryotic communities in nutrient-poor and nutrient-rich environments, respectively (Figure 4). Increasing labile soil nutrients and plant residue quality can modulate microbial communities in pre-season soils, since SOM and TN levels in postharvest soils increased during continuous cultivation with chemical fertilizers (Dong et al., 2012).

The effects of cultural practices on community variations were significant but weak (Supplementary Table 3). Previous studies reported that cropping practices show significant moderate to substantial effects on soil bacterial and fungal communities during cropping season (Ai et al., 2018; Hartman et al., 2018). The difference in the magnitude of effects of cultural practices may be related to sampling sites showing different soil physicochemical properties and microbial composition. Another possibility is the loss of soil nutrients during overwintering. A previous study reported that soil nitrogen can leach by freeze-thaw cycles (Joseph and Henry, 2008). Considering that paddy soils freeze and thaw from winter to spring, nitrogen sources may loss from soil during overwintering, suggesting reduced effect of fertilization on microbes. Further studies regarding nutrient loss during overwintering and soil microbial communities may address this question.

Plants produce and excrete root exudates to recruit microbial communities. Root exudates consist of labile carbon and organic nitrogen sources (sugars, sugar alcohols, phenolics, and amino acids). A previous study showed that copiotrophs are positively correlated with the amount of carbon in root exudates and that resource competition between microorganisms creates a root environment favoring oligotrophic populations (Maloney et al., 1997). Copiotrophic microbes could become dormant when carbon sources are depleted during plant senescence (Kjelleberg et al., 1987), and break dormancy when sufficient nutrients are restored. Nitrogen fertilizer could stimulate the exudation of carbon-containing compounds from plant roots (Mergel et al., 1998). It can be inferred that soil microbial communities might undergo higher nutrient availability under fertilized conditions than under non-fertilized conditions during plant growth. Continuous application of fertilizer could increase soil organic carbon level by increasing the quantity of plant residues (Dong et al., 2012), supporting the growth of copiotrophs when plants are absent. This finding suggests that continuous fertilization might lead to the dominance of active copiotrophs in both bulk and rhizosphere soils during plant growth. As we used an amplicon-based approach to investigate microbial communities, whether predicted copiotrophs and oligotrophs are in dormant or active state in examined soils could not be easily determined. By considering dormant and active states in microbial communities, the soil ecology of microbial trophic lifestyles may be further clarified.

Fertilization affected microbial networks in several ways, leading to the loss of community stability. The numbers of nodes and edges, as well as complexity of microbial networks decreased, whereas negative associations increased under fertilized conditions in both nutrient-poor and nutrient-rich soil environments (Supplementary Figures 15, 16 and Supplementary Table 11). Studies of grassland soil (Wagg et al., 2019) and wheat roots suggest that network complexity may be correlated with community functionality and resilience (Banerjee et al., 2019). Considering that microbial communities under non-fertilized conditions showed more associations (more nodes and edges) and greater complexity with fewer negative associations than fertilized conditions, community stability might be greater under natural conditions without soil nutrient manipulation. High nutrient concentrations led to more negative interactions between species and exclusion of more species from the community, resulting in a loss of biodiversity decreasing the stability of microbial communities under *in vitro* conditions (Ratzke et al., 2020). These findings suggest that the increase in nutrients might lead to an increase in negative associations in soil microbial communities under the field conditions. Further meta-omics (metagenome, metatranscriptome, and metaproteome)-based approaches will help to identify significant relationships between functional diversity, functional stability, competitive associations among microbes, and fertilization.

Archaea were important participants in microbial associations in the soils analyzed in the present study. Archaea significantly contributed to the degree and betweenness centrality values of multi-kingdom associations, despite their low numbers (Supplementary Figure 18). Archaea are abundant in rice paddy soils (Liesack et al., 2000) and acidic sediments (Korzhenkov et al., 2019). Archaea in rice paddy soils play central roles in carbon (methanogenic archaea) (Liesack et al., 2000) and nitrogen (ammonia-oxidizing archaea) (Wang et al., 2009) cycling. Our network analysis revealed that methanogenic archaea were significantly and positively associated with bacteria in methane cycling, iron reduction, and methanotrophy. Methanogenic archaea also had positive associations with fungal saprotrophs (Supplementary Table 13). Adding rice straw and root residues could enhance the growth of the archaeal methanogens, *Methanosarcinaceae* and *Methanosaetaceae*, respectively (Peng et al., 2008). The positive associations between archaeal methanogens and fungal saprotrophs predicted in the soil network analysis suggest that the decomposition activities of fungal saprotrophs may affect methanogenic activity. This finding indicates that archaeal communities potentially mediate soil functions through interactions with different microbial kingdoms in rice paddy soils. In addition, we suggest that methane production in and emission from rice paddy soils might depend on not only bacteria and archaea but also fungi. However, archaea-related microbial associations require further analyses, including assessment of the distribution of functional genes or proteins involved in microbial metabolism.

To classify oligotrophs and copiotrophs in soil microbial communities, previous studies conducted an independent assessment based on culture-independent and culture-dependent approaches. For example, the copy number of rRNA operon (rrn) gene has been used to identify microbial trophic lifestyles of bacteria and archaea (Kearns and Shade, 2018; Bledsoe et al., 2020). Culture-dependent assessment using media having different levels of carbon sources was performed to investigate copiotrophs and oligotrophs from culturable microbes (Senechkin et al., 2010). These independent assessments may redeem the limitation of correlation-based classification of oligotroph and copiotroph. In the present study, we classified microbial trophic lifestyles based on the correlations between contents of SOM or TN and relative abundances of OTUs without an independent assessment due to the following reasons. First, rrn copy number-based classification is not currently available for fungi. Since we aimed to investigate trophic lifestyles of bacterial, archaeal, and fungal communities parallelly, we could not perform this approach. Secondly, a culture-dependent approach cannot fully cover trophic lifestyle at the community level because the culturability of soil microbes is very low (0.3% of total soil microbes) (Amann et al., 1995). Another difficulty is that short partial sequences of OTUs do not match one-to-one with full-length marker gene sequences of culturable isolates. For example, different species that have an identical sequence in a specific variable region can match with the same OTU based on sequence similarity. This difficulty is similar to that in the identification and taxonomic classification of OTUs with short sequence reads using reference databases cataloging sequences of full-length marker genes (Yadav et al., 2019). Due to these difficulties, we did not assess oligotrophs and copiotrophs based on a culture-dependent approach. However, we identified that OTUs belonging to *Cladosporium, Mucor*, *Glomeromycota*, *Penicillium*, *Roseobacter*, and *Rhizobiaceae* are also predicted as the identical class that previously reported taxa as oligotroph or copiotroph (Ho et al., 2017). Taking together, our prediction on oligotroph and copiotroph is not perfectly accurate but tends to reflect the results of previous reports.

## Conclusion

Our findings contribute to the conceptual advancement of how soil nutrients shape the composition and multi-kingdom associations of bacterial, archaeal, and fungal communities. Our findings also underscore that the increase in nutrient dependency of microbiotas by continuous fertilization could lead to a loss of soil sustainability. Continuous fertilization may deplete oligotrophic functional members and enrich a few copiotrophic members, leading to high nutrient dependency in the microbial communities. This dependency of microbial communities is referred to as “nutrient addiction,” a synonym for “fertilizer addiction,” which is defined as a negative feedback loop requiring continuous fertilizer applications to meet the desired yield under continuous soil nutrient loss (Dolgin, 2018). The nutrient-addicted state demands a high level of nutrients to maintain community functions. An insufficient supply of nutrients may lead to the collapse of microbial community compositions and functions, resulting in dysbiosis-like soil sickness. Future research should assess the relationship between functional changes, nutrient dependency of microbial communities, and loss of soil sustainability. More broadly, our study will help fill a major knowledge gap in the microbial ecology of soils before cropping season.

## Data Availability Statement

The datasets presented in this study can be found in online repositories. The names of the repository/repositories and accession number(s) can be found below: https://www.ncbi.nlm.nih.gov/, PRJNA603275. Raw input files and all the codes used for statistical analyses in this study are available at: https://github.com/hyunkim90/Soil_microbiome_precultivation_stage.

## Author Contributions

HK and Y-HL conceived and designed the study, discussed and interpreted the results, and contributed to the writing of the manuscript. HK, KL, and JJ carried out the soil collection and analyzed the data. All authors read and approved the final manuscript.

## Conflict of Interest

The authors declare that the research was conducted in the absence of any commercial or financial relationships that could be construed as a potential conflict of interest.

## Publisher’s Note

All claims expressed in this article are solely those of the authors and do not necessarily represent those of their affiliated organizations, or those of the publisher, the editors and the reviewers. Any product that may be evaluated in this article, or claim that may be made by its manufacturer, is not guaranteed or endorsed by the publisher.

## Abbreviations

aOTU: archaeal OTU
bOTU: bacterial OTU
CC: Chuncheon
CF: conventional farming
CJ: Cheongju
CL: clay loam
daOTU: differentially abundant OTU
DG: Daegu
Fer: fertilized fields
fOTU: fungal OTU
IS: Iksan
JJ: Jinju
L: loam
MY: Miryang
NF: no fertilizers
NJ: Naju
Non-fer: non-fertilized fields
NP: no pesticides
RA: relative abundance
SC: silty clay
SCL: silt clay loam
Silt L: silt loam
SL: sandy loam
UF: University Farm (Suwon)
YS: Yesan.
