# Supplementary material for: Compositional Shift of Bacterial, Archaeal, and Fungal Communities Is Dependent on Trophic Lifestyles in Rice Paddy Soil
Source: Front Microbiol. 2021 Sep 1;12:719486. doi: 10.3389/fmicb.2021.719486 (PMC8440912; doi:10.3389/fmicb.2021.719486)
Supplement: Supplementary file 1 [file Data_Sheet_1.zip › Data Sheet 1.pdf]

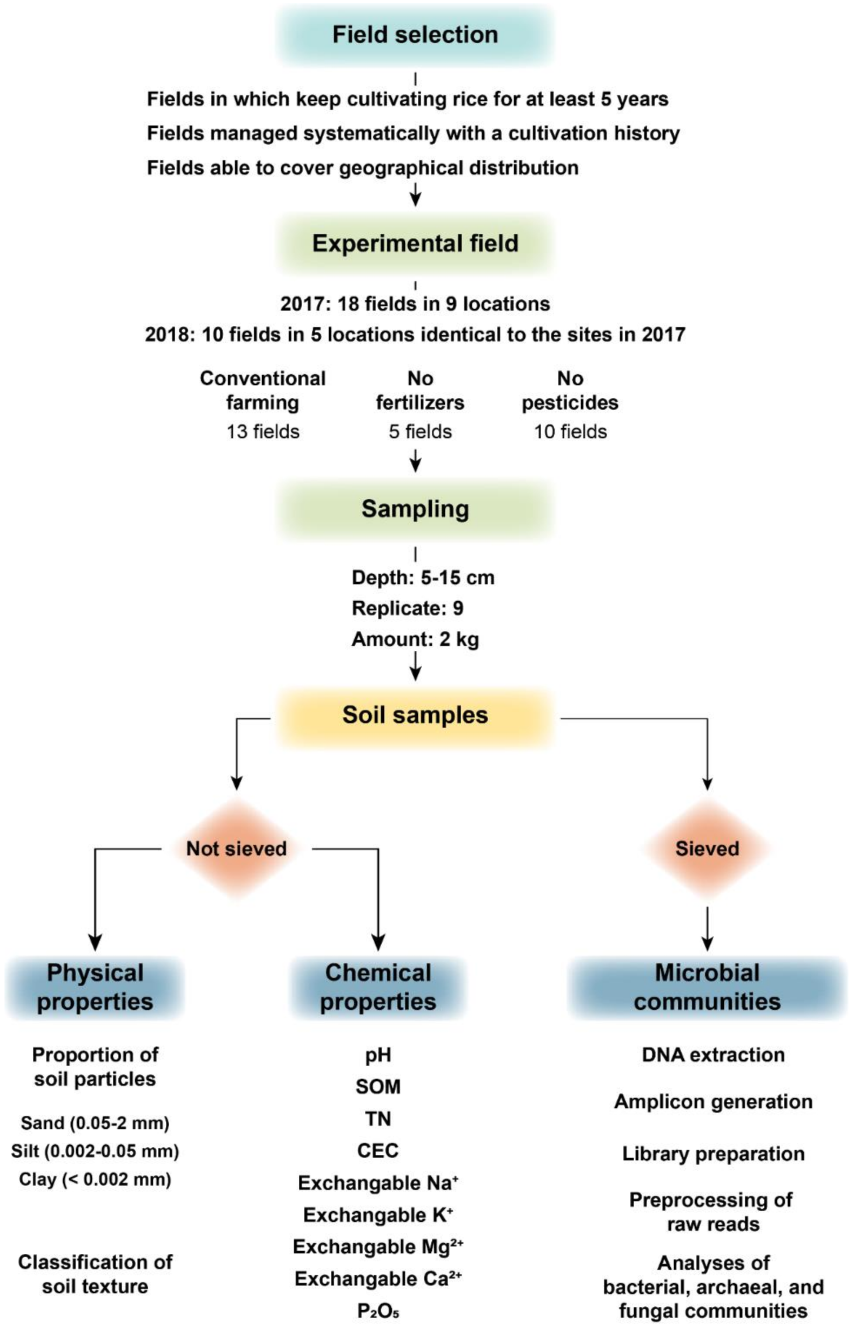

Supplementary Figure 1. Experimental scheme of this study. In this study, fields which met three criteria were selected to investigate pre-season soils of rice paddies. Based on the criteria, 18 fields were selected in 2017. Among the fields, 10 fields which were identical to the fields investigated in 2017 were investigated again in 2018. Nine replicates per a field were acquired. Total 252 soil samples were collected in 2017 and 2018. Collected soil samples were sieved for extracting DNAs from soils. Soils which were not sieved were used for investigating physical and chemical properties. Using data on microbiota (bacterial, archaeal, and fungal community) and soil physicochemical properties, correlations between microbiota and soil properties were examined. Details on entire experiments and analyses were described in Materials and Methods.

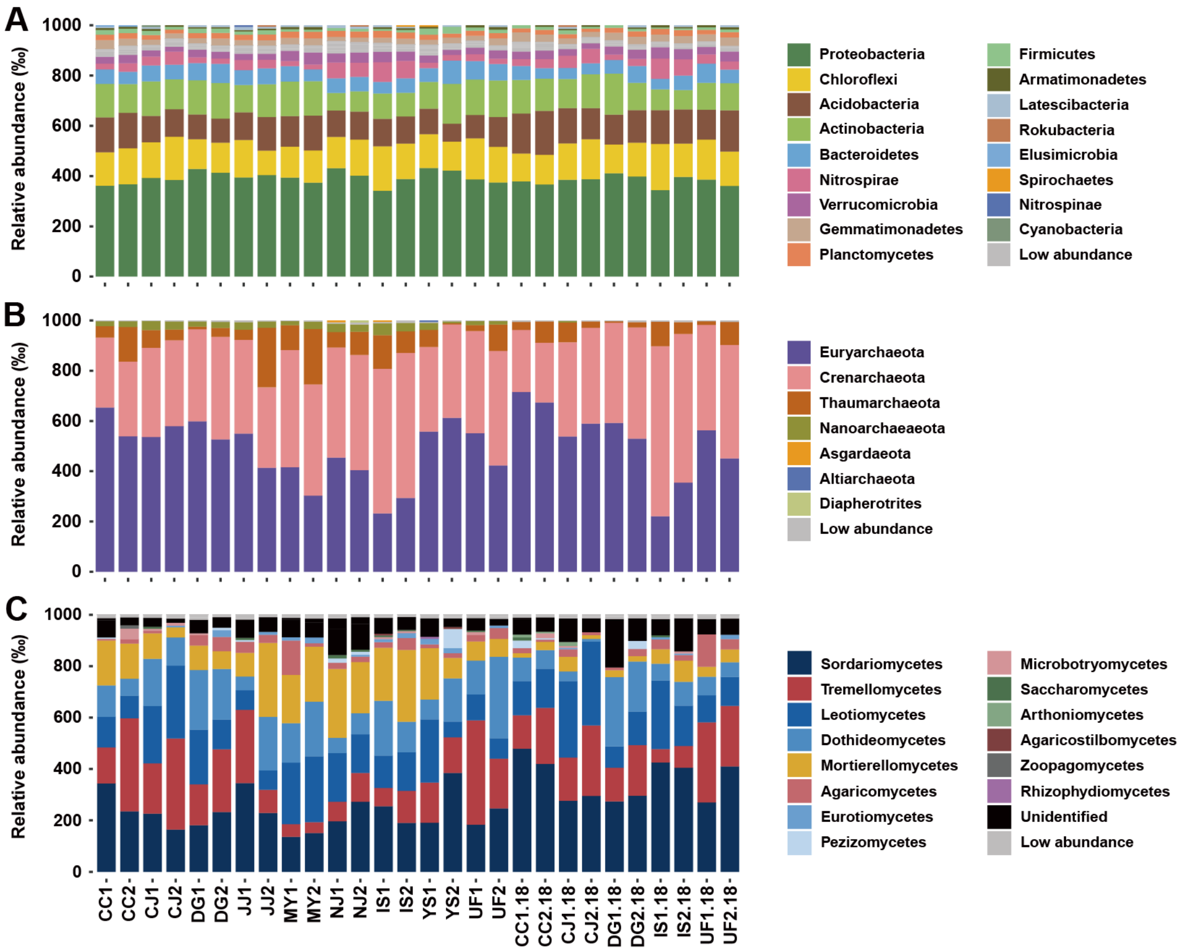

Supplementary Figure 2. Composition of bacterial, archaeal, and fungal communities in each field. The relative abundances of the bacterial (**A**), archaeal (**B**), and fungal (**C**) communities were investigated at the phylum and class levels, respectively. Less abundant taxonomic groups (taxa which relative abundances were less than 5 ‰ of each sample) are indicated in gray. Unidentified taxa are indicated in black. The fields investigated in 2018 are labeled with the suffix ".18". CC, Chuncheon; CJ, Cheongju; DG, Daegu; IS, Iksan; JJ, Jinju; MY, Mirayng; NJ, Naju; UF, University Farm (Suwon); YS, Yesan.

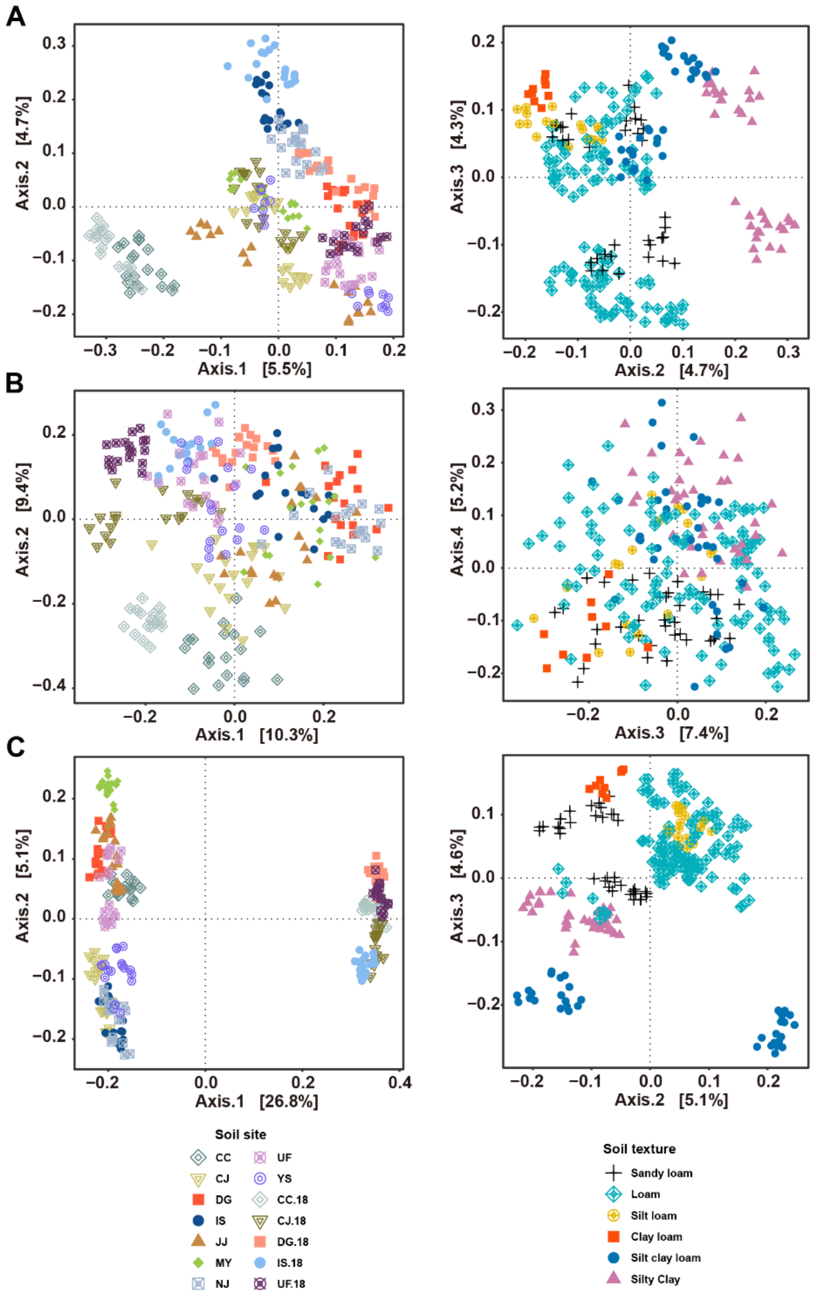

Supplementary Figure 3. Unconstrained principal coordinate analysis (PCoA) on soil bacterial (A), archaeal (B), and fungal (C) communities. To estimate the compositional distances among samples, Bray-Curtis distances were estimated. For this, cumulative sum scaling (CSS)/log-transformed reads were used. Samples were grouped by soil site (left panel) and soil texture (right panel). In the left panel, the fields investigated in 2018 are labeled with the suffix ".18".

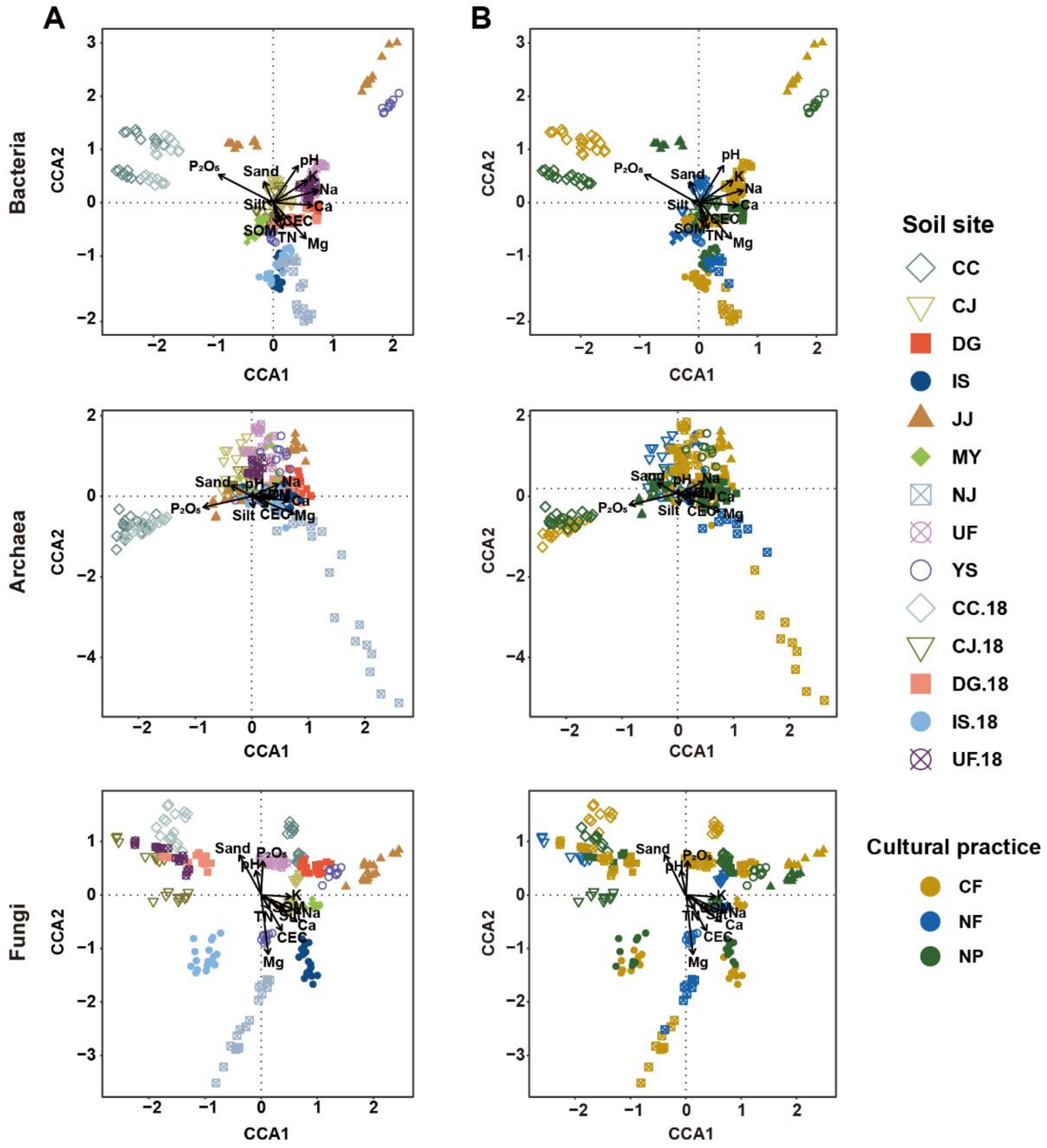

Supplementary Figure 4. Canonical correspondence analysis on bacterial, archaeal, and fungal communities in rice paddy fields. The CSS-normalized and log-transformed OTU tables were used as inputs for bacterial, archaeal, and fungal amplicon data. The fields investigated in 2018 are labeled with the suffix ".18". Samples were grouped by paddy fields (**A**) and cultural practices (**B**). Upper, middle, and bottom panels indicate bacterial, archaeal, and fungal communities, respectively. In the CCA plots, the direction of arrows indicates the direction where the level of a particular factor increases. The length of arrows represents the effect size of each edaphic factor on community variances. SOM, soil organic matter; TN, total nitrogen; CEC, cation-exchange capacity; P_2O_5, phosphate; K, potassium; Mg, magnesium; Na, sodium; Ca, calcium; CC, Chuncheon; CJ, Cheongju; DG, Daegu; IS, Iksan; JJ, Jinju; MY, Mirayng; NJ, Naju; UF, University Farm (Suwon); YS, Yesan.

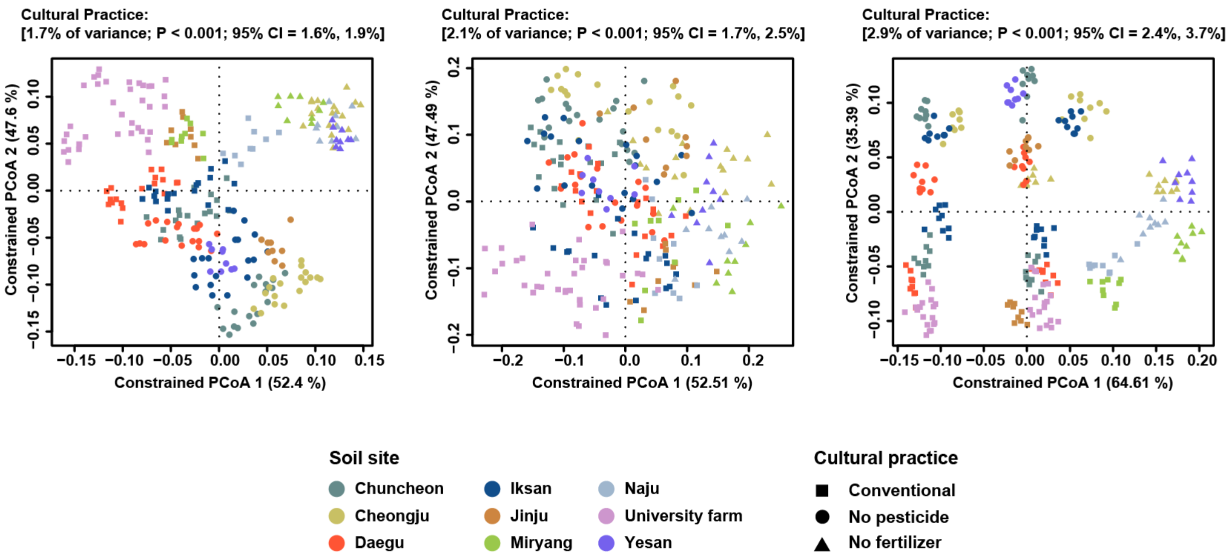

Supplementary Figure 5. Constrained principal coordinate analysis (constrained PCoA) on bacterial, archaeal, and fungal communities in rice paddy fields. Variations of bacterial (left), archaeal (middle), and fungal (right) communities constrained by cultural practices. Colors represent soil sites, and each cultural practice is indicated as square (conventional farming; n = 117), circles (No pesticide; n = 90), and triangles (No fertilizer; n = 45). Cumulative sum scaling (CSS)/log-transformed reads were used to calculate Bray–Curtis distances.

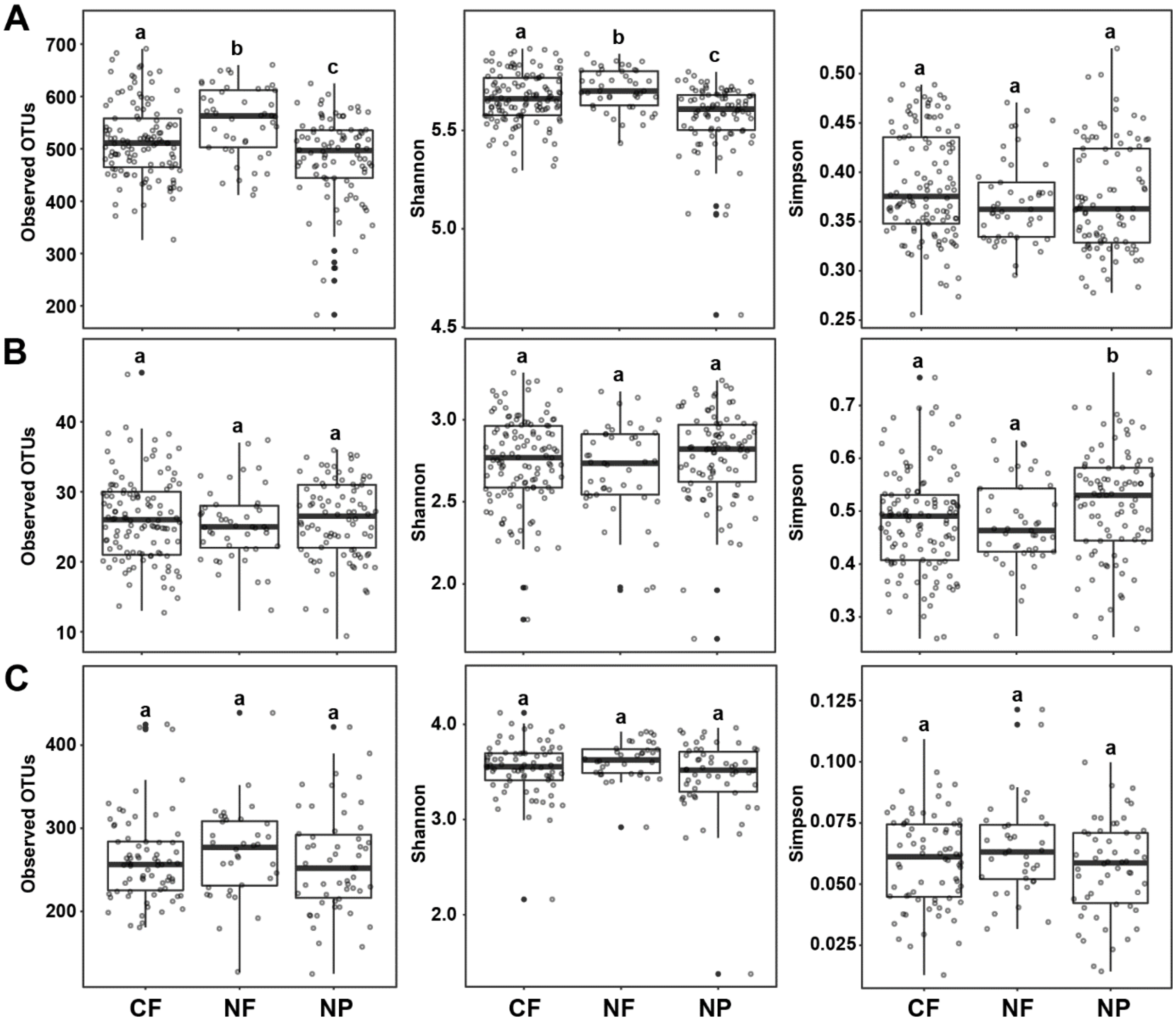

Supplementary Figure 6. Alpha diversity of bacterial (A), archaeal (B), and fungal communities (C) of rice paddy soils according to cultural practices. Richness and evenness of soil microbial communities are investigated with observed OTUs, Shannon, and Simpson indices. Alpha diversity indices are estimated based on rarefied OTU tables. Statistically significant differences between group means of relative abundance were determined by Kruskal-Wallis test followed by Dunn's test ($P < 0.05$). CF, conventional farming (n=117); NF, no fertilizer (n=45); and NP, no pesticide (n=90).

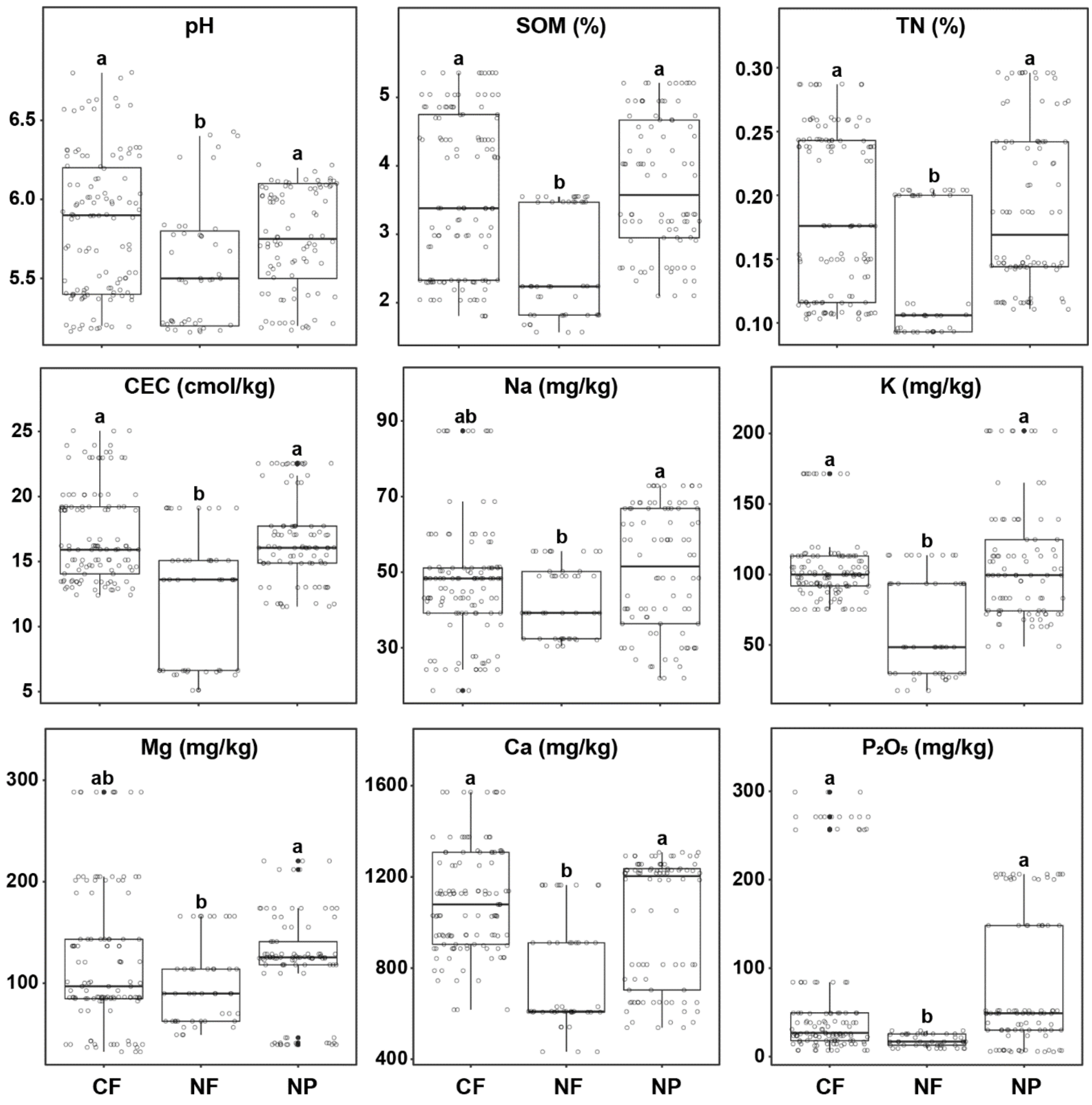

Supplementary Figure 7. Statistical analysis on soil chemical properties depending on cultural practices. Statistically significant differences between group means of observed values were determined by Kruskal-Wallis test followed by Dunn's test ($P < 0.05$). CF, conventional farming (n = 117); NF, no fertilizer (n = 45); and NP, no pesticide (n = 90). SOM, soil organic matter; TN, total nitrogen; CEC, cation-exchange capacity; P_2O_5, phosphate; K, potassium; Mg, magnesium; Na, sodium; and Ca, calcium.

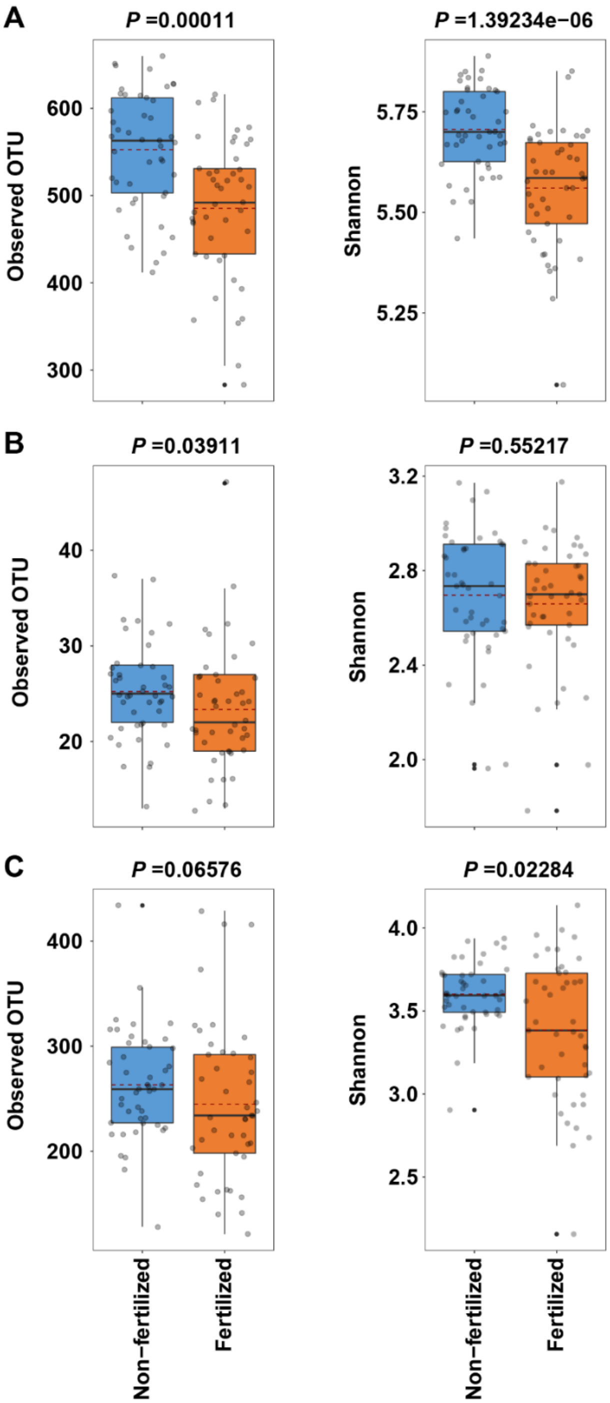

Supplementary Figure 8. Alpha diversity of bacterial (A), archaeal (B), and fungal communities (C) of rice paddy soils in non-fertilized and fertilized fields. Alpha diversity of soil microbial communities of non-fertilized and fertilized fields is investigated with observed OTUs and Shannon index. Alpha diversity indices are estimated based on rarefied OTU tables. Statistically significant differences between group means of relative abundance were determined by Wilcoxon test. Non-fertilized, non-fertilized fields (n = 45); Fertilized, fertilized fields (n = 45).

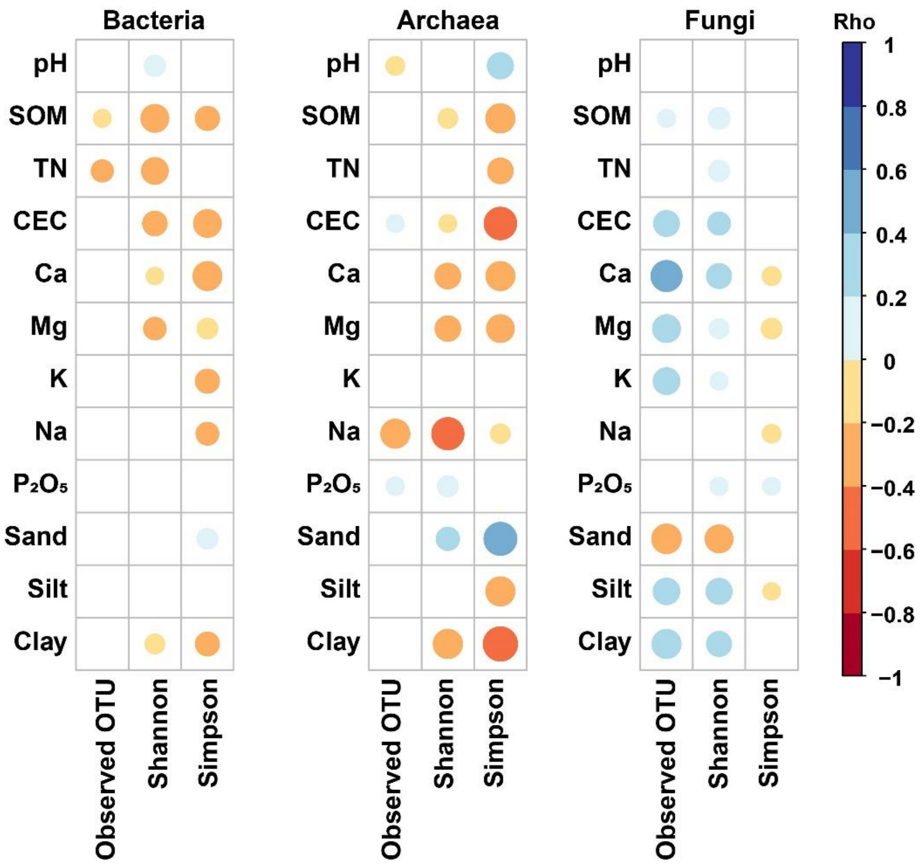

Supplementary Figure 9. Spearman correlation analysis between edaphic factors and alpha diversity indices. Significant correlations which *P*-value is under 0.05 were displayed in the plot. Color and size of circles indicate the values of correlation coefficients. Colors close to red show negative correlations, whereas colors close to blue indicate positive correlations. Shannon, Shannon index; Simpson, Simpson index; SOM, soil organic matter; TN, total nitrogen; CEC, cation-exchange capacity (number of exchangeable cations per unit dry weight); P_2O_5, phosphate; K, potassium; Mg, magnesium; Na, sodium; Ca, calcium.

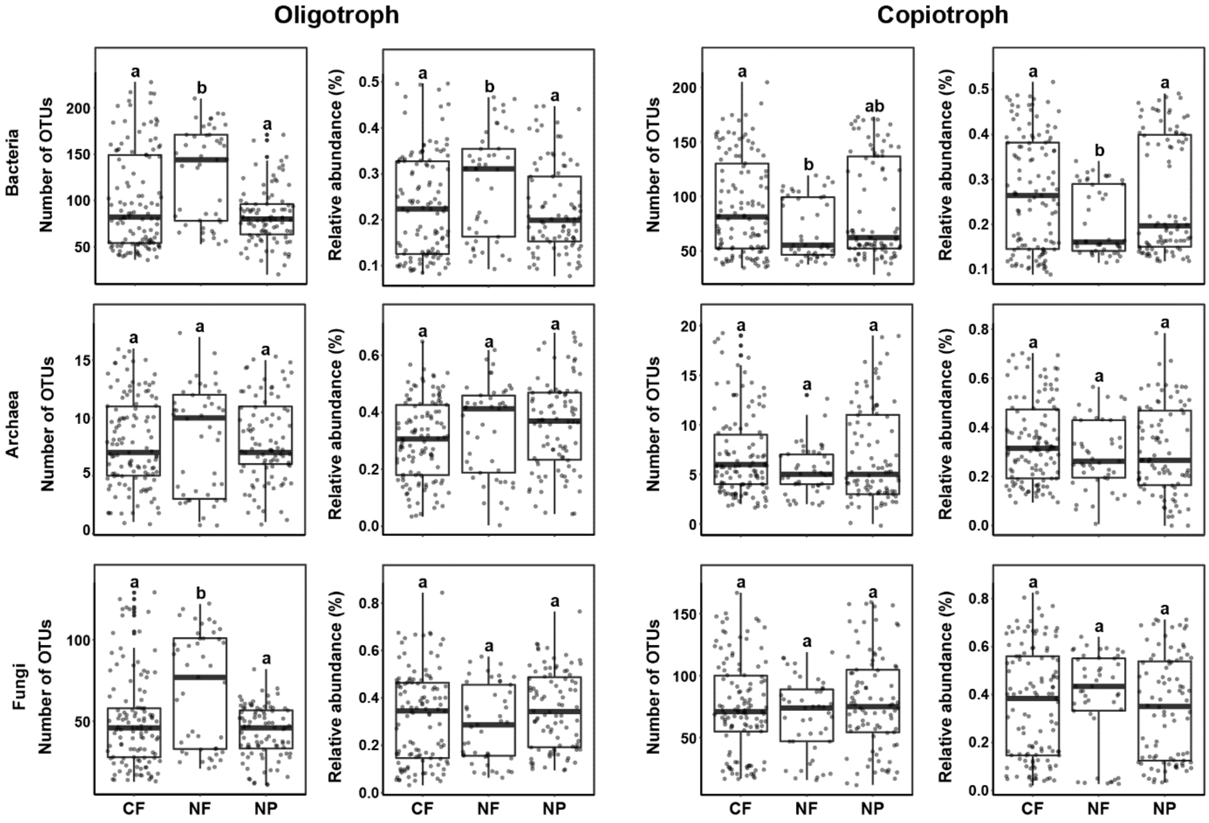

Supplementary Figure 10. Statistical analyses on OTU counts and relative abundances of putative oligo- and copiotrophs in bacterial, archaeal, and fungal communities according to cultural practices. Each box indicates cultural practices. Dots represent the numbers or relative abundances of putative oligotrophs and copiotrophs in each soil sample. The letters indicate significance of the differences. Statistically significant differences between group means of each value were determined by Kruskal-Wallis test followed by Dunn’s test ($P < 0.05$).

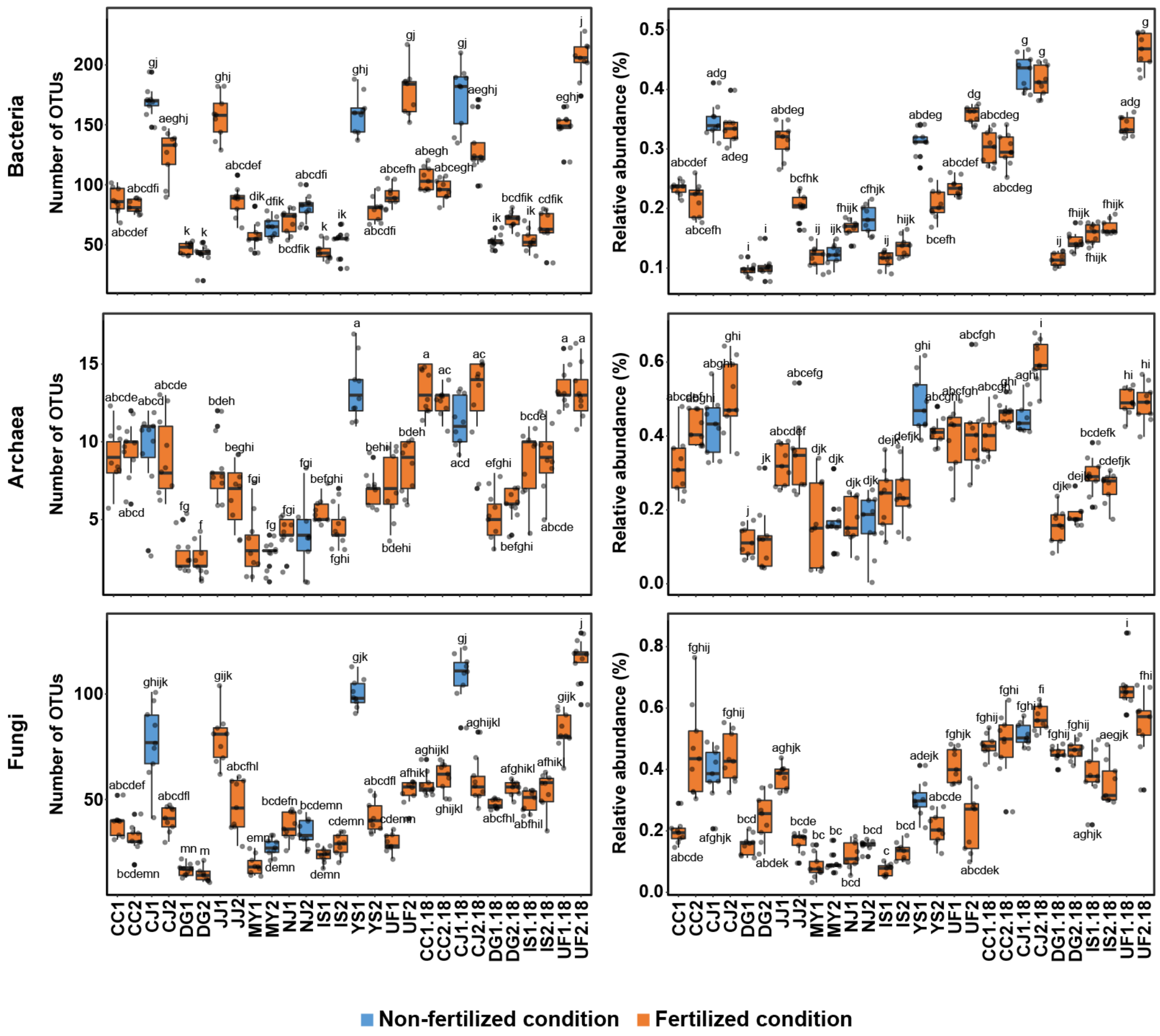

Supplementary Figure 11. Statistical analyses on OTU counts and relative abundances of putative oligotrophs in bacterial, archaeal, and fungal communities in paddy fields. Each box indicates the experimental fields. Fertilized and non-fertilized fields are colored by orange and light blue, respectively. Dots represent the numbers or relative abundances of putative oligotrophs and copiotrophs in each soil sample. The letters indicate significance of the differences. Statistically significant differences between group means of each value were determined by Kruskal-Wallis test followed by Dunn's test ($P < 0.05$).

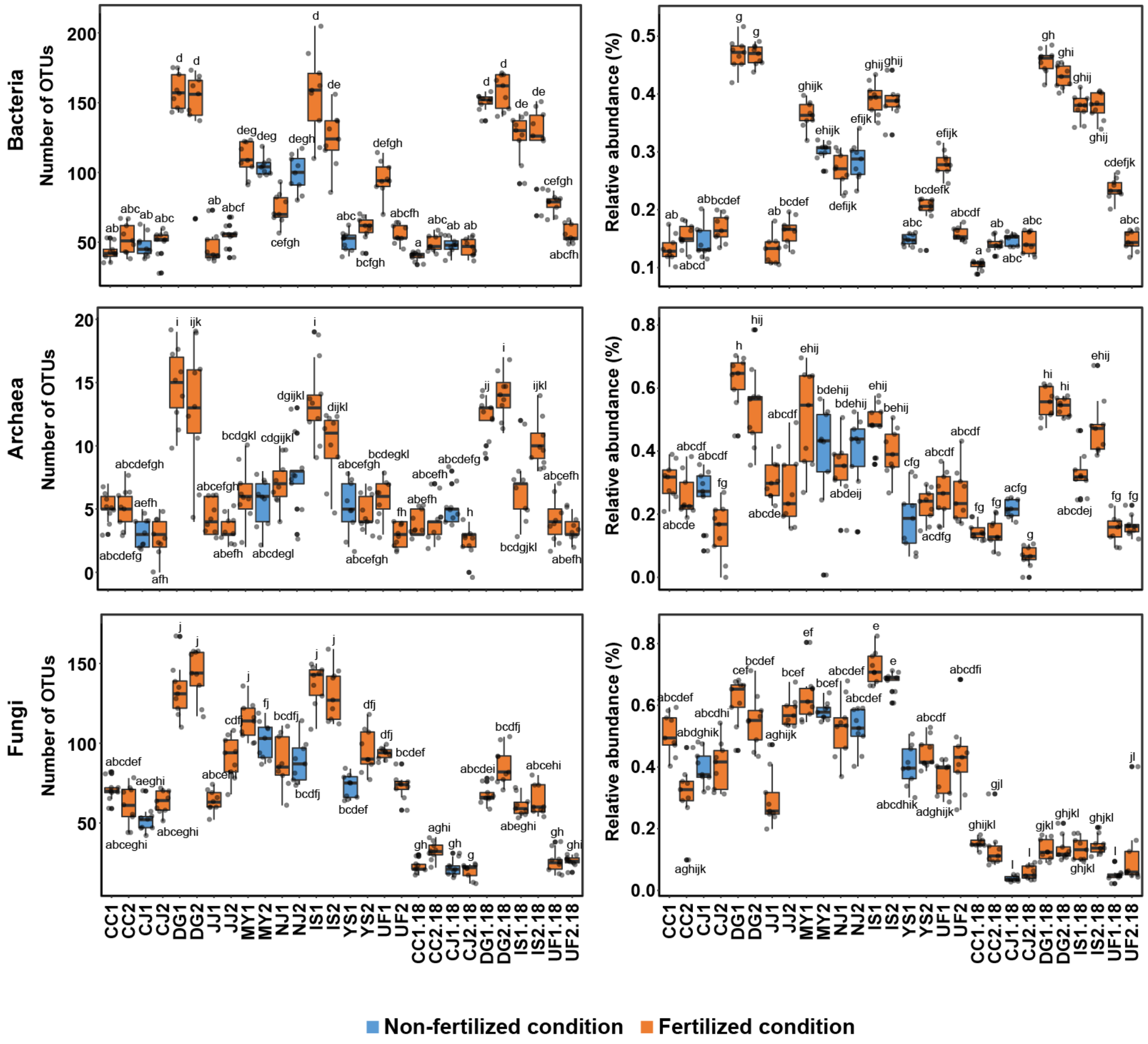

Supplementary Figure 12. Statistical analyses on OTU counts and relative abundances of putative copiotrophs in bacterial, archaeal, and fungal communities in paddy fields. Each box indicates the experimental fields. Fertilized and non-fertilized fields are colored by orange and light blue, respectively. Dots represent the numbers or relative abundances of putative oligotrophs and copiotrophs in each soil sample. The letters indicate significance of the differences. Statistically significant differences between group means of each value were determined by Kruskal-Wallis test followed by Dunn's test ($P < 0.05$).

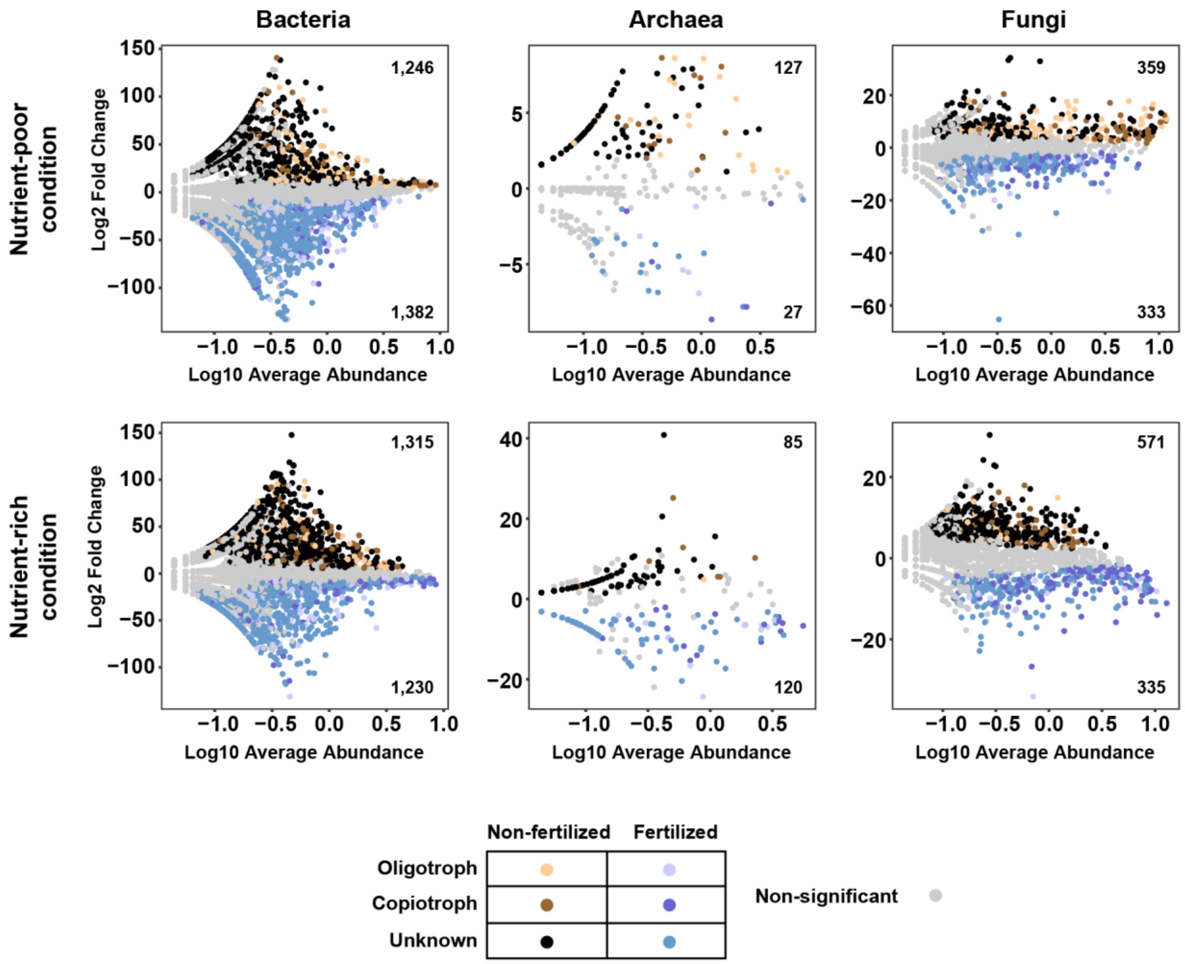

Supplementary Figure 13. Differentially abundant OTUs in nutrient-poor and –rich conditions in soil microbial communities. The comparison was made using a zero-inflated Gaussian distribution mixture model on the CSS normalized OTU tables followed by a moderated t-test and a Bayesian approach. Data from all nine replicates of each field were used. This test revealed 1,246 bacterial, 127 archaeal, and 359 fungal OTUs that were significantly enriched in non-fertilized soils in the nutrient-poor environment (above 2 folds [$\log_2$ Fold change > 1], $P < 0.05$). A total of 1,382 bacterial, 27 archaeal, and 333 fungal OTUs that were significantly enriched in fertilized soils in the nutrient-poor environment (above 2 folds [$\log_2$ Fold change < -1], $P < 0.05$). In the nutrient-rich environment, 1,315 bacterial, 85 archaeal, and 571 fungal OTUs that were significantly enriched in non-fertilized soils in the nutrient-poor environment, whereas a total of 1,230 bacterial, 120 archaeal, and 335 fungal OTUs that were significantly enriched in fertilized soils. Each dot indicates individual OTUs. Dots are colored by their designation based on the classification of trophic lifestyles and enrichment patterns. Non-significant OTUs are indicated as gray dots.

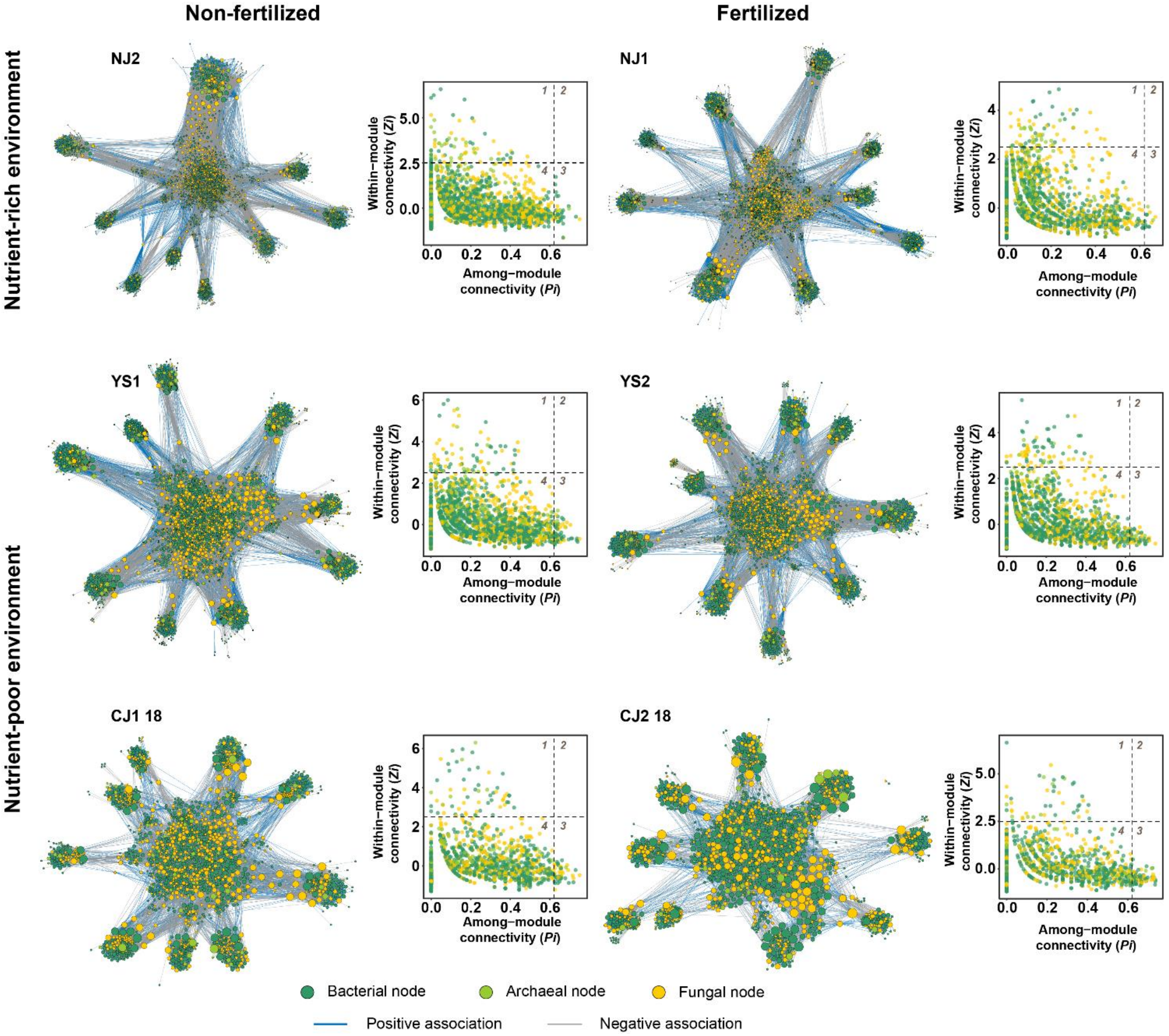

Supplementary Figure 14. Microbial networks and hub nodes of soil microbial communities of non-fertilized and fertilized conditions in the nutrient-poor and -rich environments. Co-occurrence-based networks of microbial OTUs detected in the fields managed with different fertilization regimes in the nutrient-poor and –rich environments (left panel). Each node corresponds to an OTU, and edges between nodes correspond to either positive (blue) or negative (gray) correlations inferred from OTU abundance profiles using the SparCC method ($P < 0.05$, correlation coefficient < -0.6 or > 0.6). OTUs belonging to different microbial kingdoms have different color codes (Bacteria, green; Archaea, light green; Fungi, yellow), and node size reflects their degree centrality. Hub OTUs of microbial networks in each condition (right panel). Dashed lines indicate the threshold estimated by the within-module connectivity (*Zi*) and among-module connectivity (*Pi*) values which roles of nodes are discriminated. The numbers of quadrant indicate the roles of nodes in networks. 1, module hub (*Zi* > 2.5 and *Pi* < 0.62); 2, network hub (*Zi* > 2.5 and *Pi* > 0.62); 3, connector (*Zi* < 2.5 and *Pi* > 0.62); 4, peripheral (*Zi* < 2.5 and *Pi* < 0.62).

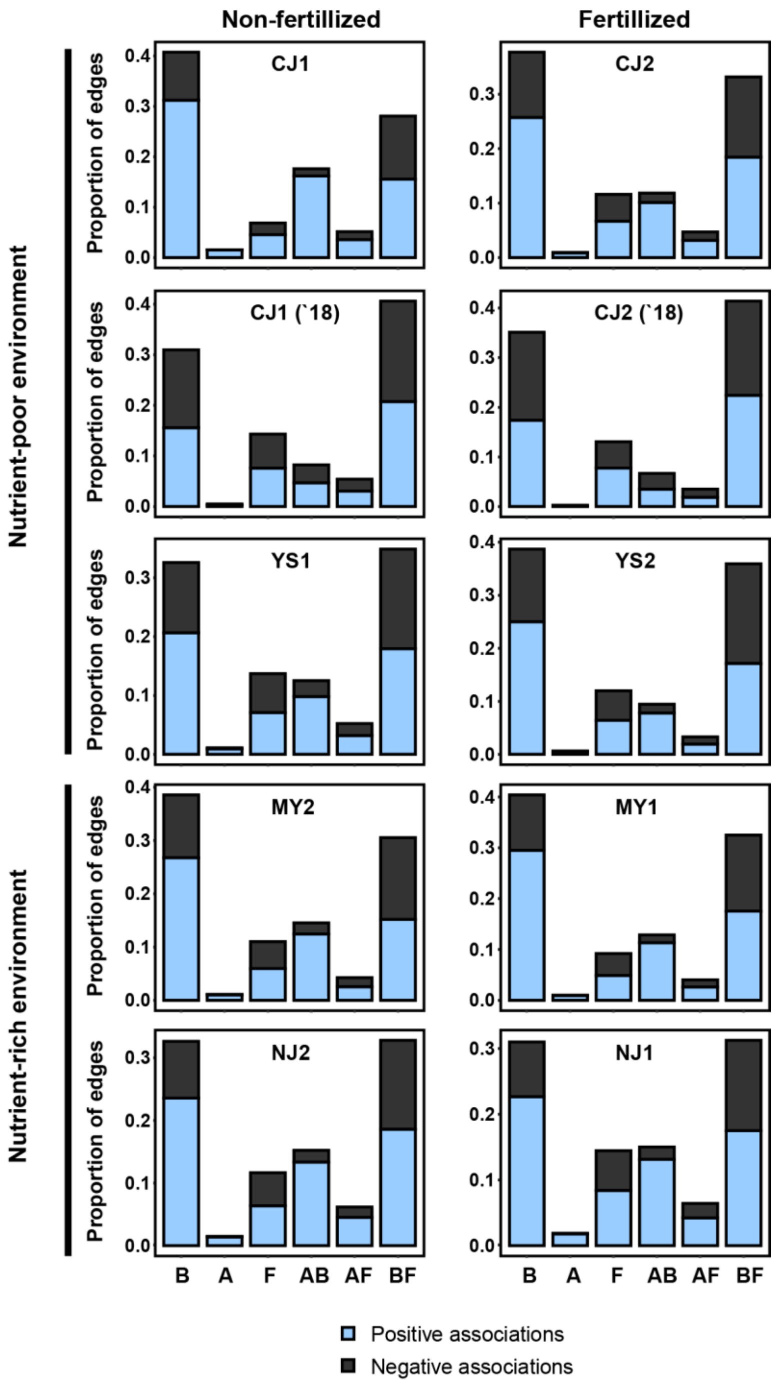

Supplementary Figure 15. Proportion of positive and negative associations of microbial networks in nutrient-poor and –rich environments. Positive and negative associations were estimated based on the correlation coefficients of each association (correlation coefficient > 0.6, positive; correlation coefficient < -0.6, negative). In the bar graph, colors indicate positive (light blue) and negative (black) associations. The numbers of positive and negative associations in networks are available in Supplementary Table 11.

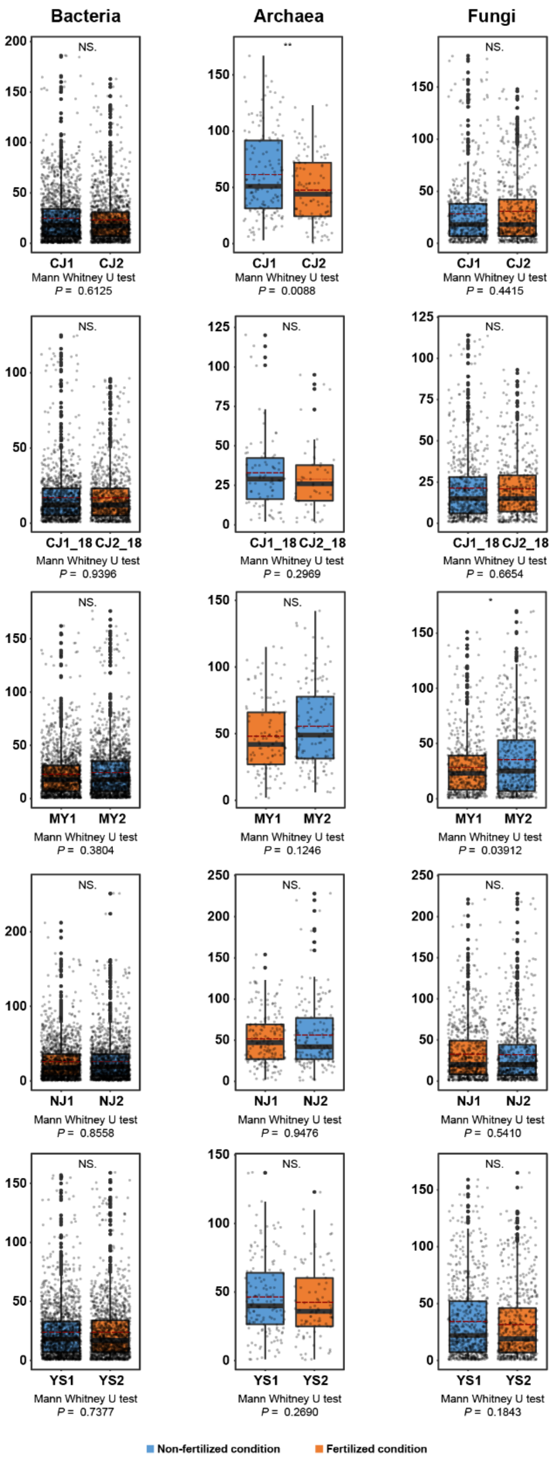

Supplementary Figure 16. Pairwise comparison on degree between the networks of non-fertilized and fertilized field at each location. Degree centrality of each microbial community was compared along with the soil sites. Each field is colored by fertilization regimes (orange, fertilized; blue, non-fertilized). Dashed red lines indicate the values of mean degree. Statistically significant differences between dissimilarity distances were determined by Mann Whitney U test (***, $P < 0.001$; **, $P < 0.01$; *, $P < 0.05$; NS, $P > 0.05$).

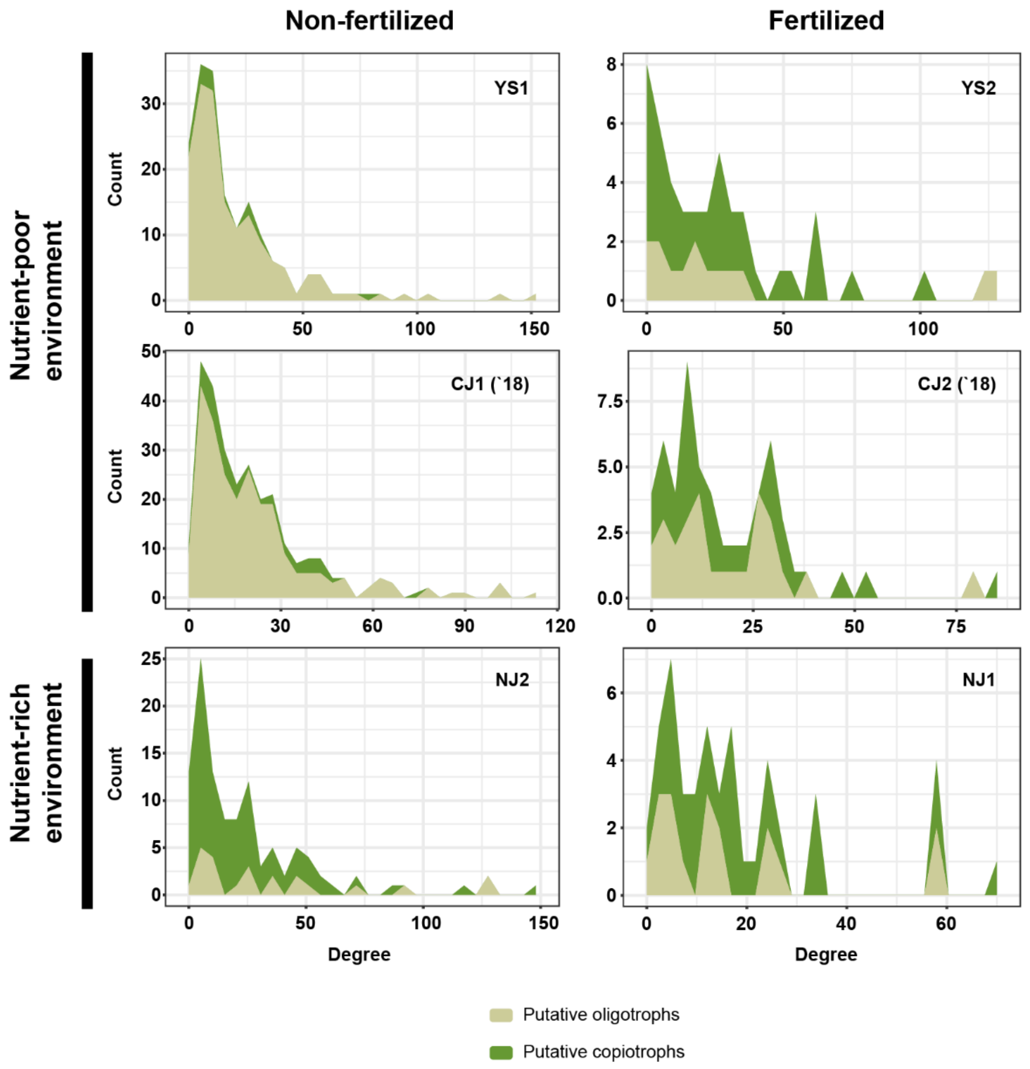

Supplementary Figure 17. Degree centrality of putative oligo- and copiotrophs in microbial networks of nutrient-poor and –rich environments. Degree indicates the number of associations shared by each node in a network. Numbers of putative oligotrophs and copiotrophs showing specific degree centrality in the microbial networks of soil sites which are not included in Figure 5 are displayed. Each color indicates putative oligotrophs (light green) and copiotrophs (dark green) in each microbial network.

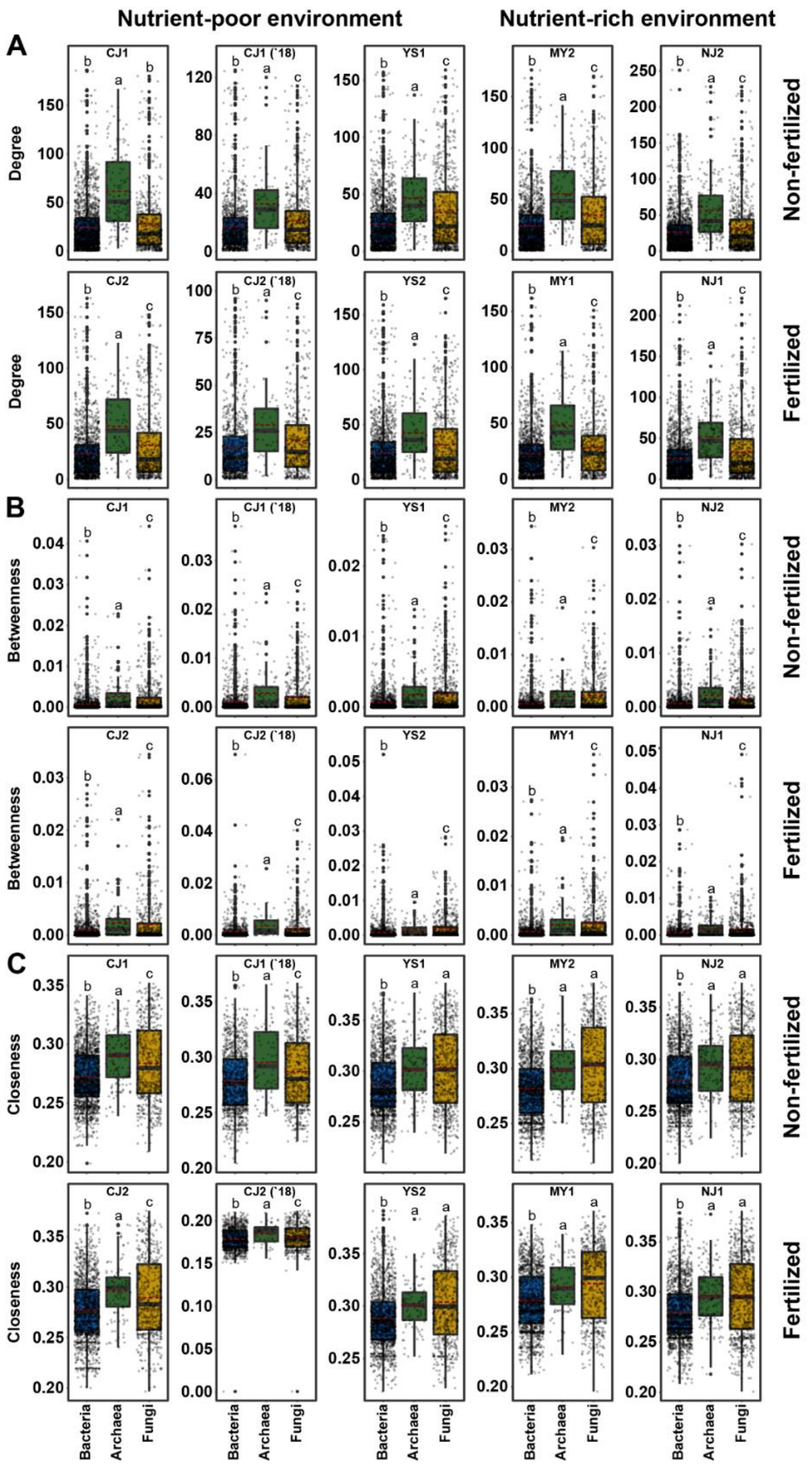

Supplementary Figure 18. Node centrality of the microbial networks of paddy fields. (**A**) degree. (**B**) betweenness centrality. (**C**) closeness centrality. The red dashed lines indicate the mean values of network centrality of each network. The letters indicate significance of the differences. Statistically significant differences between group means of each centrality were determined by Kruskal-Wallis test followed by Dunn's test ($P < 0.05$).

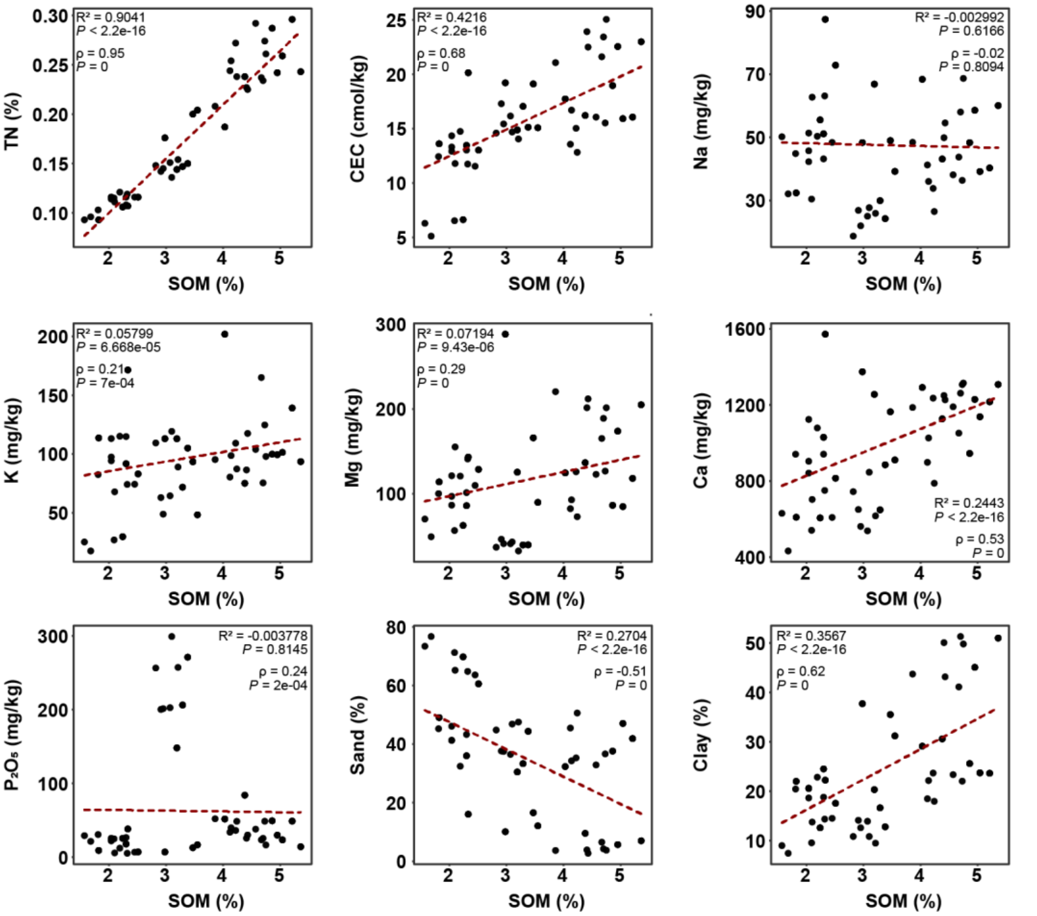

Supplementary Figure 19. Relationship between soil organic matters and other soil physicochemical properties. Correlations among soil physicochemical properties were estimated using a linear regression and Spearman rank sum correlation. The results of each statistical analysis are indicated in the plots (linear regression; R^2 with *P*-vaules; Spearman's rank correlation; ρ (rho) with *P*-values). The red dashed lines indicate trend lines of each plot.
